# Global Research Trends on Major Pathogenic Enteric Viruses (1990–2024): A Bibliometric Analysis of Epidemiology, Transmission, and Public Health Impact

**DOI:** 10.3390/pathogens14090938

**Published:** 2025-09-16

**Authors:** Mohammad Alotaibi, Hanan Al-Khalaifah, Assia Bouhoudan

**Affiliations:** 1 Food Security Program, Environment and Life Science Research Centre, Kuwait Institute for Scientific Research, Safat 13109, Kuwait; hkhalifa@kisr.edu.kw; 2 Laboratory of Research and Development in Engineering Sciences, Faculty of Sciences and Techniques of Al-Hoceima, Abdelmalek Essaadi University, Tetouan 93000, Morocco; assia.bouhoudan@uae.ac.ma

**Keywords:** enteric viruses, bibliometric analysis, epidemiology, diagnostics, public health, one health

## Abstract

Pathogenic enteric viruses are a leading cause of gastroenteritis-related mortality worldwide. However, the architecture of this research field remains poorly quantified. This bibliometric analysis provides a comprehensive overview of 35 years of global scientific output on major enteric viruses, such as rotavirus, norovirus, astrovirus, sapovirus, and non-polio enteroviruses, to map trends, methodological developments, and geographic disparities. We conducted a systematic search of PubMed and Scopus (1990–2024), identifying 10,017 records. After deduplication and eligibility screening, a final corpus of 8320 publications was analyzed using Bibliometrix (Biblioshiny 5.0) in R (version 4.3.0) and VOSviewer (Version 1.6.20). We found that scientific production grew steadily (CAGR = 5.84%), reaching its peak in 2021. The field is characterized by profound thematic and geographic disparity: rotavirus dominated the literature (56.3% of publications), followed by norovirus (30.8%), while other viruses were severely underrepresented (<9% each). Geographically, output was highly concentrated, with the top five countries (the USA, China, Japan, India, and Brazil) producing 92.4% of the publications. In contrast, high-burden regions, such as sub-Saharan Africa and Latin America, contributed only 7.6%. Genomic sequencing gained prominence, being cited in over 26.2% of publications from 2020 to 2024, reflecting a methodological shift accelerated by the application of wastewater-based epidemiology during the COVID-19 pandemic. In conclusion, while genomic tools and environmental monitoring are transforming enteric virus research, its progress is hampered by deep and persistent inequalities. These include a narrow focus on rotavirus and a significant disparity between regions with high disease burdens and those with high research outputs. Closing this gap requires targeted investments in equitable collaboration, local genomic capacity, and integrated public health interventions combining vaccination, WASH, and One Health strategies.

## 1. Introduction

Pathogenic enteric viruses, especially rotavirus and norovirus, remain the leading cause of acute gastroenteritis worldwide [1], responsible for an estimated 151,714 deaths among children under five in 2019 [2] and disproportionately affecting low-income regions [3]. Norovirus is responsible for approximately 699 million cases and 200,000 deaths annually [4,5]. Despite advances in vaccination and water, sanitation, and hygiene (WASH) infrastructure, these viruses continued to burden healthcare systems [6]. The transmission of enteric viruses occurs via fecal–oral routes, contaminated water, food, direct person-to-person contact, and environmental persistence [7,8]. Their resistance to disinfectants (especially norovirus) complicates control efforts [9]. Geographic and climatic factors significantly influence patterns: in temperate regions, rotavirus hospitalizations declined by 40–50% following vaccine introduction, whereas rates remain elevated in tropical settings [10,11]. Norovirus outbreaks typically peak in winter and result in significant economic losses [12,13]. Emerging concerns include zoonotic transmission pathways (e.g., hepatitis E virus from livestock reservoirs) [14,15]. The varied incubation periods and at-risk populations complicate prevention efforts (Table 1).

Despite numerous clinical and epidemiological studies, a systematic mapping of the global research architecture on these viruses is lacking. To our knowledge, no global bibliometric study has examined the thematic evolution, collaboration trends, and regional disparities over a 35-year period. This study aims to fill this gap, guiding research priorities, funding, and public health policies.

Although individual reviews have examined clinical epidemiology or focused on single agents (e.g., rotavirus only) [1,27], to our knowledge, no previous study has simultaneously mapped the bibliometric landscape of multiple enteric viruses, examined thematic shifts between diagnosis and prevention, and quantified regional collaboration over a period of more than three decades. By filling these gaps, our work provides the first comprehensive analysis of the evolving global research priorities and networks in enteric virus research.

Against this background, this study aimed to systematically characterize global research on pathogenic enteric viruses from 1990 to 2024 by analyzing publication trends over time, identifying key thematic focuses in epidemiology, transmission, diagnostics, and prevention, and highlighting critical research gaps to recommend future directions for policy, funding, and capacity building.

## 2. Materials and Methods

### 2.1. Study Design (PICOS Criteria)

**Figure 1:**
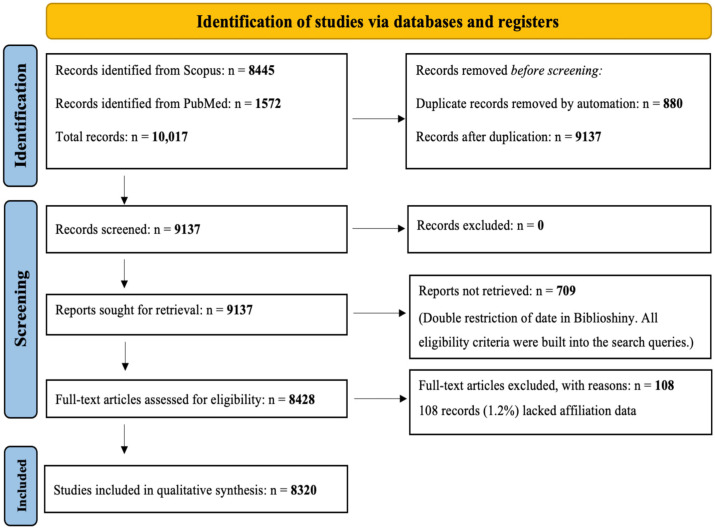
PRISMA flow diagram. Workflow diagram summarizing the bibliometric data collection process. Records removed before screening include duplicates removed automatically by R (v4.3.0). No manual screening exclusions were applied, as all eligibility criteria were incorporated into the database search strategy. Additional exclusions occurred during retrieval (*n* = 709, due to double date restriction in Biblioshiny) and at the eligibility stage (*n* = 108, records lacking affiliation data).

This study is a descriptive and cartographic bibliometric analysis conducted in accordance with the PRISMA 2020 guidelines for the transparency of systematic reviews (the PRISMA format is used as the reporting structure, but no protocol was registered on PROSPERO, which does not cover this type of analysis) [28], the full checklist is provided in Supplementary Materials (Supplementary File S1), since the flow diagram is presented in Figure 1. The methodological objectives follow the PICOS framework adapted for bibliometric reviews:

- Population (P): The scientific literature on the major pathogenic enteric viruses defined in this study was published between 1 January 1990, and 31 December 2024. We included publications addressing enteric viruses of significant public health importance, including rotavirus (family *Sedoreoviridae*), norovirus, sapovirus, and astrovirus (Caliciviridae, Astroviridae), as well as adenovirus (Adenoviridae). Within the Picornaviridae family, we considered enteroviruses (non-polio enteroviruses such as coxsackievirus and echovirus), as well as human parechoviruses (genus Parechovirus) and Aichi virus (genus Kobuvirus), both of which are increasingly recognized as enteric pathogens. For clarity, parechoviruses and Aichi virus were treated as distinct categories, not as subgroups of enteroviruses.
- Intervention (I): Bibliometric studies (i.e., analyses of publication trends, authorship, citations, keyword co-occurrence).
- Comparator (C): Not applicable.
- Outcomes (O): Research trends, including publication volume, geographic distribution, collaboration networks, thematic evolution, and methodological shifts in diagnostics and prevention.
- Study designs (S): Original research articles and review papers.

### 2.2. Information Sources and Search Strategy

The databases searched were PubMed (Medline) and Scopus, chosen for their comprehensive coverage of the biomedical literature and the availability of metadata suitable for bibliometric analyses. The searches were performed on 1 February 2025.

We designed the search strategy to capture all relevant literature on pathogenic enteric viruses defined in the PICOS criteria (Section 2.1). We employed a systematic search strategy that combined general and specific terminology related to enteric viruses (including search terms for both “enterovirus” and other picornavirus genera (e.g., “parechovirus”, “Aichi virus”) to capture the full scope of enteric picornaviruses; however, for taxonomic clarity in the manuscript we treat non-polio enteroviruses (NPE) as members of the genus Enterovirus (family Picornaviridae) and list human parechoviruses (genus Parechovirus) and Aichi virus (genus Kobuvirus) separately.), their epidemiology, diagnosis, and public health impact. Searches were conducted in PubMed Title/Abstract and MeSH fields, as well as in Scopus TITLE-ABS-KEY fields. The strings were pilot-tested on a subset and refined to ensure optimal coverage.

#### Search Details

- Keywords and MeSH:
  o General: “enteric viruses”, “gastroenteritis”, “diarrhea”, “vomiting”, “dehydration”, “fecal-oral transmission”, “public health”, “epidemiology”, “transmission”, “diagnosis”, “prevention”, “vaccination”, “sanitation”, “hygiene”.
  o Specific: “rotavirus”, “norovirus”, “enteric adenovirus”, “astrovirus”, “sapovirus”, “viral gastroenteritis”, “waterborne diseases”, “foodborne diseases”, “outbreaks”, “clinical manifestations”, “molecular diagnostics”, “antigen-based tests”, “electron microscopy”, “oral rehydration therapy”, “vaccine efficacy”, “global burden”, “low-income countries”, “high-income countries”.
  o MeSH Terms: “Enterovirus Infections”[Mesh], “Gastroenteritis”[Mesh], “Diarrhea”[Mesh], “Vomiting”[Mesh], “Dehydration”[Mesh], “Fecal-Oral Route”[Mesh], “Public Health”[Mesh], “Epidemiology”[Mesh], “Disease Transmission, Infectious”[Mesh], “Diagnosis”[Mesh], “Prevention & Control”[Mesh], “Vaccination”[Mesh], “Sanitation”[Mesh], “Hygiene”[Mesh], “Rotavirus”[Mesh], “Norovirus”[Mesh], “Adenoviruses, Human”[Mesh], “Astroviridae”[Mesh], “Sapovirus”[Mesh].
- Boolean Example: (“enteric virus*” OR “gastroenter*” OR diarrh*) AND (“fecal-oral transmiss*” OR “public health” OR epidemiolog*).
- Wildcards Examples: “enteric virus*”, “enterovir*”, “gastroenter*”, “diarrh*”, “vomit*”, “dehydrat*”, “fecal-oral transmiss*”, “public health*”, “epidemiol*”, “transmiss*”, “diagnos*”, “prevent*”, “vaccinat*”, “sanitat*”, “hygien*”.
- Timeframe: 1990–2024 (search performed 1 February 2025).
- Search Queries:
  o Scopus Query:TITLE-ABS-KEY( (  rotavirus OR norovirus OR “enteric adenovirus” OR “adenovirus F40” OR “adenovirus F41”  OR astrovirus OR sapovirus  OR enterovir* OR coxsackievirus* OR echovirus*  OR “Aichi virus” OR kobuvirus OR parechovirus* OR picornaviridae ) AND (  “viral gastroenter*” OR gastroenteritis OR diarrh* OR vomit* OR dehydrat* OR “fecal-oral” OR waterborne OR foodborne ) AND ( epidemiol* OR transmiss* OR diagnos* OR “molecular surveillance” OR “genomic sequenc*” OR prevent* OR vaccinat* OR “antiviral therap*” OR WASH OR sanitati* OR “climate change” ))AND ( LIMIT-TO(DOCTYPE, “ar”) OR LIMIT-TO(DOCTYPE, “re”) )AND ( EXCLUDE(SUBJAREA, “VETE”) OR EXCLUDE(SUBJAREA, “AGRI”) )AND (PUBYEAR > 1989 AND PUBYEAR < 2025)AND ( LIMIT-TO(LANGUAGE, “English”) OR LIMIT-TO(LANGUAGE, “French”) )
  o PubMed Query:( (  rotavirus[Title/Abstract] OR rotavirus[MeSH Terms]  OR norovirus[Title/Abstract] OR norovirus[MeSH Terms]  OR “enteric adenovirus”[Title/Abstract] OR “adenovirus F40”[Title/Abstract] OR “adenovirus F41”[Title/Abstract] OR “Adenoviridae”[MeSH Terms]  OR astrovirus[Title/Abstract] OR “Astroviridae”[MeSH Terms]  OR sapovirus[Title/Abstract]  OR enterovirus[Title/Abstract] OR “Enterovirus Infections”[MeSH Terms]  OR coxsackievirus[Title/Abstract] OR echovirus[Title/Abstract]  OR “Aichi virus”[Title/Abstract] OR kobuvirus[Title/Abstract]  OR parechovirus[Title/Abstract] OR “Parechovirus”[MeSH Terms] OR “human parechovirus”[Title/Abstract]  OR picornaviridae[Title/Abstract] ) AND (  “gastroenteritis”[Title/Abstract] OR “Gastroenteritis”[MeSH Terms]  OR diarrh*[Title/Abstract] OR “Diarrhea”[MeSH Terms]  OR vomit*[Title/Abstract] OR “Dehydration”[MeSH Terms]  OR “fecal-oral route”[MeSH Terms] OR “waterborne diseases”[MeSH Terms] OR “foodborne diseases”[MeSH Terms] ) AND (  epidemiology[MeSH Terms] OR “disease transmission”[MeSH Terms] OR diagnosis[MeSH Terms]  OR “molecular surveillance”[Title/Abstract] OR “genomic sequencing”[Title/Abstract]  OR prevention[MeSH Terms] OR vaccination[MeSH Terms] OR “antiviral agents”[MeSH Terms]  OR sanitation[MeSH Terms] OR “climate change”[MeSH Terms] ))AND (english[Language] OR french[Language])AND (“1990/01/01”[Date - Publication]: “2024/12/31”[Date - Publication])AND (Humans[Mesh]).

### 2.3. Eligibility Criteria

We applied strict criteria to ensure the relevance and quality of the included publications.

- Inclusion Criteria: Research articles and reviews in English and French on the epidemiology, transmission, diagnosis, prevention, and public health of enteric viruses. We acknowledge that this language restriction may underrepresent research published in languages other than English, such as Spanish, Portuguese, Chinese, or in non-indexed regional journals, which is a potential source of selection bias.
- Exclusion Criteria: Studies should directly relate to epidemiology or public health and focus on human enteric viruses. Exclude animal-only research, non-English/French papers, and non-peer-reviewed sources such as conference proceedings, non-peer-reviewed book chapters, and editorials.
- Focus Specification: To maintain a focus on gastroenteritis, the analysis of enterovirus-related publications was restricted to those reporting on gastrointestinal manifestations or fecal viral excretion.
- Age: No age restrictions were applied; pediatric and adult studies were included.
- Co-infections: Studies reporting coinfections (viral, bacterial, and/or parasitic agents) were included only if virus-specific data could be unequivocally extracted (e.g., if results were stratified by pathogen or if viral outcomes were reported separately). If viral data could not be separated from non-viral outcomes, the article was excluded from virus-specific thematic analyses but documented in the selection journal.

### 2.4. Study Selection Process

Our systematic search identified 10,017 records from Scopus (*n* = 8445) and PubMed (*n* = 1572). After automatically removing 880 duplicates, 9137 unique records were retained for analysis.

Due to the bibliometric nature of this review, which aims to analyze publication trends and networks across a large body of literature, all primary eligibility criteria (document type, language, time period, and subject) were incorporated directly into the database search queries. This approach, standard in bibliometrics, favors exhaustiveness and reproducibility over subjective manual exclusion at the screening stage, as our research questions focus on macro-trends rather than individual study results.

However, two post-search technical exclusions were necessary. Seven hundred nine records could not be processed in the Biblioshiny software (Biblioshiny 5.0) due to a redundant publication date filter during export and were therefore excluded from the analysis. Second, an additional 108 records (1.2%) were excluded during the data cleaning and deduplication phase (see Section 2.6.2) due to the absence of essential affiliation metadata, a prerequisite for conducting authorship and collaboration analyses.

To validate the relevance of our automated search strategy, we performed quality control by manually reviewing a random sample of 5% (*n* = 457) of the deduplicated records. This inspection confirmed that the search filters successfully retrieved publications corresponding to the intended scope, according to established large-scale bibliometric protocols [29]. The final corpus for bibliometric analysis comprised 8320 publications. The study selection process is summarized in the PRISMA flowchart (Figure 1).

Given the bibliometric nature of this analysis, which focuses on macro-trends rather than individual study results, no formal risk of bias assessment was performed on the included records.

### 2.5. Data Extraction and Management

#### 2.5.1. Export and Import

- PubMed: Retrieved PMID, title, abstract, author names, affiliations, publication date, keywords, MeSH terms, and citation metadata (where available).
- Scopus: Retrieved EID/DOI, title, abstract, author names, affiliations, publication date, keywords, citation counts, journal name, and other bibliometric fields.
- All exports were saved as CSV and imported into RStudio (v4.3.3) using bibliometrix, readr, or data.table.

#### 2.5.2. Deduplication and Cleaning

- Priority matching by DOI, when available; otherwise, exact title, first author, and year. Via distinct() and dplyr (v1.1.0) to remove precise duplicates.
- Standardized author names (“LastName Initials”), harmonized institution and country names (ISO standards).
- Institutions: Cleaned common variants/spelling differences (e.g., “Univ. of X” vs. “University of X”).
- Flagged records missing key metadata fields (e.g., no publication year, no affiliation data). A total of 108 records were excluded at this stage due to missing affiliation data, as noted in Section 2.4.
- All preprocessing steps, counts at each stage, and any manual corrections were logged in R scripts for reproducibility.
- During the pre-processing steps, 709 records that could not be retrieved during Biblioshiny export (as noted in Section 2.5) were logged and excluded. Then, 108 records (1.2% of the evaluated records) were excluded due to missing key metadata (specifically, affiliation data), and zero records were flagged for manual correction of import errors.

### 2.6. Subset and Thematic Filtering

#### 2.6.1. Regional Subsets

- West Africa (Ghana, Nigeria, Senegal, Côte d’Ivoire, Mali, Burkina Faso, Benin, and Togo). Any record with at least one author affiliation in these countries was included; multi-affiliation papers were counted in each relevant subset but tracked to avoid double-counting when aggregating global metrics (204 records).
- Southern Africa (Botswana, Namibia, South Africa, Zimbabwe, Zambia, Malawi) with the same inclusion logic as above (309 records).

#### 2.6.2. Risk-Factor Keywords

- Terms: “sanitation,” “water,” “population density,” “zoonotic” (and variants via wildcard truncation if relevant, e.g., ‘sanitat*’, ‘zoonot*’). Automated search in title/abstract/keywords flagged 7422 articles; each was manually validated.

### 2.7. Automated Epidemiological Extraction

- Targets: Incidence, mortality, and morbidity estimates mentioned in titles/abstracts.
- Method: Regex patterns capturing numeric-per-population expressions; ~10% randomly checked and refined iteratively. Results are reported descriptively.

### 2.8. Bibliometric Analyses

- Productivity trends: Yearly counts; CAGR (compound annual growth rate) with 95% CIs calculated per standard formulas.
- Key contributors:
  o Author-, institution-, and country-level metrics: total publications and average citations per document.
- Impact Metrics: H-index, total/average citations by author/institution/country based on the collected dataset snapshot (full counting).
- Journal ranking: By publications count and impact factor (sourced from Journal Citation Reports for the relevant year; details in script).
- Software:
  o R/bibliometrix and Biblioshiny (v5.0.1) [30] for summary metrics.
  o VOSviewer (v1.6.20) [30] for co-authorship, country, and keyword co-occurrence networks (thresholds: ≥5 publications for authors; ≥10 occurrences for keywords; decadal slices).

### 2.9. Contextualization and Sensitivity Analyses

To strengthen the interpretation of our findings and address potential biases, we performed two supplementary analyses:

- Sensitivity analysis for rotavirus dominance: To assess whether the observed trends were strongly influenced by rotavirus research, we (i) excluded all publications explicitly mentioning rotavirus and (ii) applied a fractional counting method (where each article mentioning “k” viruses contributes “1/k” to each virus’s count).
- Contextualization of publication growth: to determine if the observed increase in publications in 2021 was specific to our field or part of a broader trend in biomedical publishing, we compared the annual growth of our corpus to the total number of publications indexed in PubMed each year. PubMed was chosen as the global reference because it offers free and exhaustive access to biomedical publications. The data on global PubMed production was obtained via the Entrez API.

## 3. Results

### 3.1. Study Selection

Our systematic search identified a total of 10,017 records. After automatic removal of 880 duplicates, 9137 unique records were retained for retrieval. Seven hundred nine records could not be retrieved during the Biblioshiny export (due to redundant date filters in the export settings) and were excluded from the evaluation. Thus, 8428 records were evaluated for eligibility; 108 records (1.2%) were excluded due to missing key metadata (specifically, affiliation), and no records were flagged for manual correction of import errors. The final set consisted of 8320 documents included for bibliometric analyses.

### 3.2. Descriptive Analysis

#### 3.2.1. General Overview

**Figure 2:**
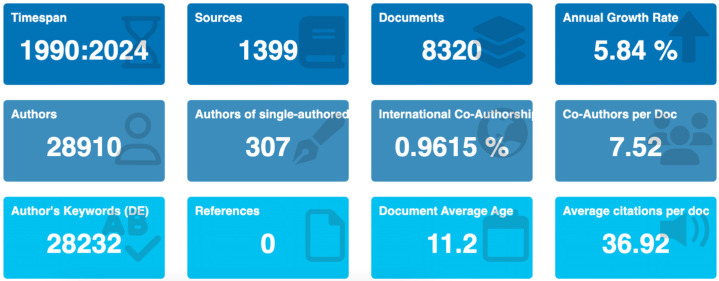
Overview and Scope of the Study. Annual evolution of scientific production (1990–2024). The upper graph displays the temporal evolution in the number of publications per year. The lower table presents key bibliometric indicators, including CAGR, collaboration rate, and article types.

After deduplication and retrieval screening, 8320 records (1990–2024) across 1399 sources were included for the descriptive analysis. Original research articles dominated (78.5%), reviews (13.2%), and other document types (8.3%) (e.g., letters, case reports). The compound annual growth rate (CAGR) was 5.84%. Annual production rose from 72 articles in 1990 to a peak of 520 articles in 2021 and 496 articles in 2024 (Figure 2). The 2021 peak aligns with the enhanced environmental surveillance (wastewater-based epidemiology) during the COVID-19 pandemic; the number of publications partially declined thereafter but remained above the levels of the early 2000s. Overall, the long-term growth, with a marked inflection after 2000, likely reflects the broader adoption of molecular diagnostics (RT-PCR, NGS) and increased surveillance funding after massive vaccine rollouts.

**Figure 3:**
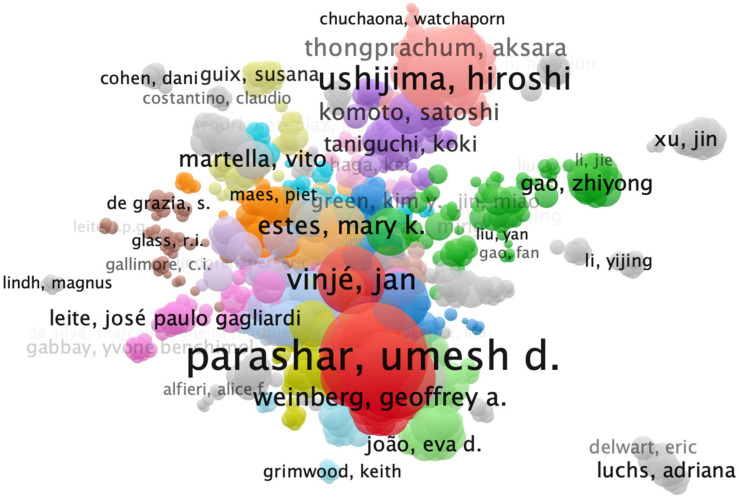
Co-authorship network of authors in enteric virus research (1990–2024). The network was constructed and visualized using VOSviewer. Nodes represent authors with a minimum of 5 publications; their size is proportional to their total number of publications. The thickness of the lines (edges) between nodes represents the strength of the co-authorship link. Colors represent distinct thematic clusters determined by VOSviewer’s clustering algorithm based on collaboration strength. The most prominent clusters are directly labeled on the map with their primary research focus, including (1: Red) Surveillance and Vaccine Impact, (2: Green) Clinical Epidemiology and Diagnostics, (3: Blue) Molecular Characterization, (4: Yellow) Environmental Transmission, (5: Purple) Vaccinology/Immunology, (10: Light Red) Rotavirus in Asia, (13: Gray) Norovirus Evolution, and (24: Slate Gray) Viral Discovery.

Co-authorship networks from VOSviewer highlighted Umesh Parashar from the U.S. Centers for Disease Control and Prevention (CDC) (*n* = 332 articles) and Roger Glass from the National Institutes of Health (NIH) (*n* = 146 articles) as pivotal contributors. The analysis also revealed structured thematic clusters, including surveillance and vaccine impact; clinical epidemiology and diagnostics; molecular characterization; environmental transmission; vaccinology/immunology; and geo-specific clusters (Figure 3).

#### 3.2.2. Geographic and Institutional Distribution

**Figure 4:**
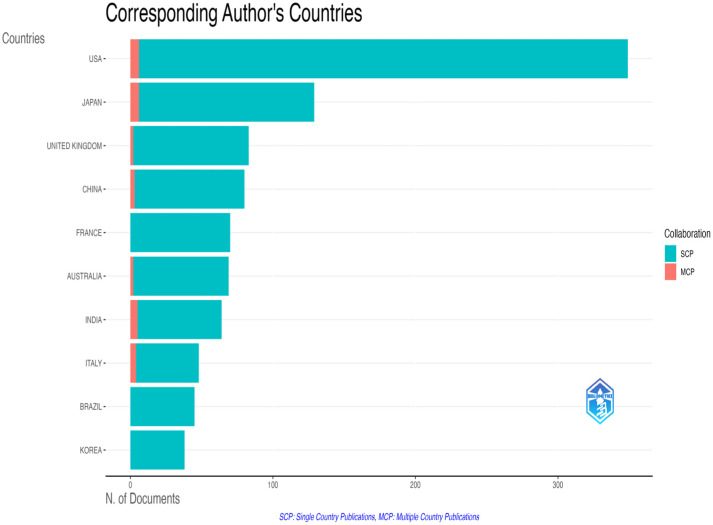
Country-level collaboration network (1990–2024). Nodes represent countries. Size and color indicate the volume of publications. Edge thickness denotes the number of internationally co-authored articles, with the most substantial ties observed between the U.S. and China.

Country contributions were highly concentrated: The United States was the most significant contributor with 3180 articles (38.2%), followed by China (1912 articles, 23.0%), Japan (1034 articles, 12.4%), India (874 articles, 10.5%), and Brazil (689 articles, 8.3%). Collectively, the top five countries accounted for 92.4% of total publications (Figure 4). The most productive institution was the Centers for Disease Control and Prevention (CDC), with 902 publications, followed by the National Center for Immunization and Respiratory Diseases (NCIRD), with 521 publications.

Beyond overall productivity, we analyzed the geographic distribution of research leadership by identifying the country of the corresponding author, a key indicator of study ownership and seniority. This revealed a significant disparity between participation and leadership. While researchers from the United States contributed to 2205 publications, they served as the corresponding author on only 349 studies (15.8% leadership share). A similar pattern was observed for other high-productivity countries: China (932 total contributions vs. 80 corresponding authorships; 8.6% leadership share), Japan (641 vs. 129; 20.1%), and the United Kingdom (460 vs. 83; 18.0%).

This analysis also revealed stark evidence of inequitable research partnerships, often referred to as ‘parachute research,’ particularly in regions with high disease burdens. For instance, while researchers from Kenya, Nigeria, and Mozambique were authors on 48, 31, and 31 studies, respectively, they held corresponding authorship on only 10 (20.8%), 2 (6.5%), and 1 (3.2%) of them, respectively (Table 2). This suggests that, although international collaboration is widespread, the direction and ownership of research remain highly concentrated in a few high-income countries.

**Figure 5:**
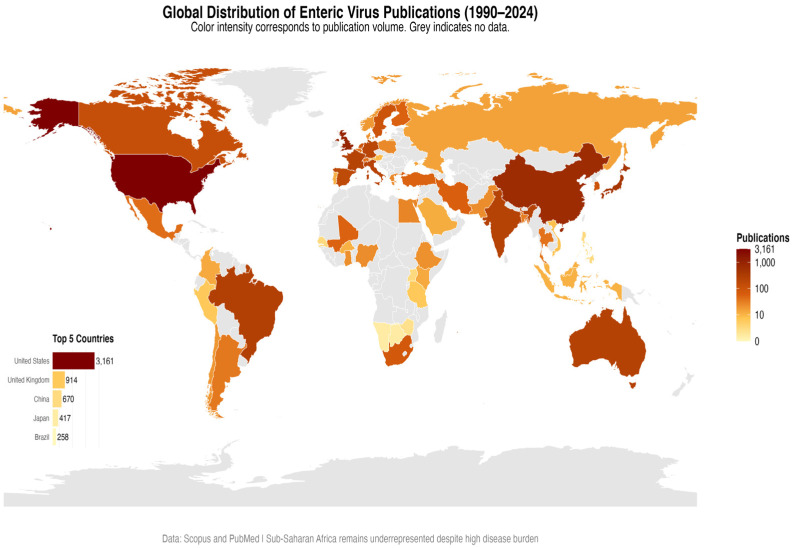
Global Distribution of Enteric Virus Publications (1990–2024). The map displays the number of publications per country, based on data from Scopus and PubMed. Color intensity (from light yellow to dark red) and node size are proportional to the publication volume. The inset bar chart details the publication counts for the five most productive countries. Most contributions came from North America, Europe, and Asia. Sub-Saharan Africa remains underrepresented despite a high disease burden.

No African country was among the top ten publishing countries, despite the continent’s high burden of enteric viral disease. Analysis of sub-regional output, however, shows that West Africa accounted for 204 publications and Southern Africa for 309 publications. South Africa and Nigeria dominate within these totals, but many countries remain underrepresented relative to disease burden and vaccine impact (Figure 5).

**Figure 6:**
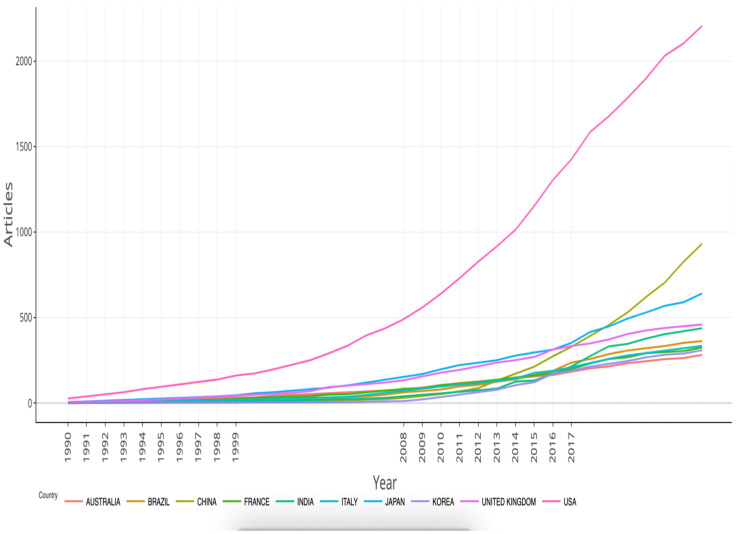
Evolution of scientific production by country (1990–2024). Cumulative number of publications per country (1990–2024). The U.S., China, and Japan exhibit the highest output. China has shown rapid growth since 2005.

China’s output rose from zero articles in 1990 to become the second most productive country by 2024 (Figure 6). The U.S. maintained both quantitative and qualitative leadership, with an average citation rate of 69 citations per article (15.3 for China).

**Figure 7:**
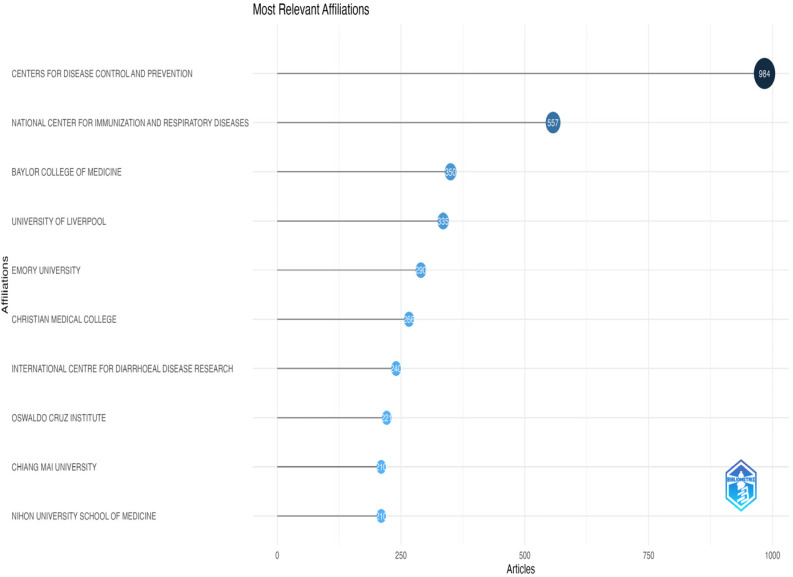
Most productive institutions (1990–2024). Bar graph listing top institutions by publication volume. Leading institutional contributors (e.g., Centers for Disease Control and Prevention—CDC; Evandro Chagas Institute; National Center for Immunization and Respiratory Diseases) are identified with their publication counts to highlight institutional productivity and influence in the field.

Among institutions, the CDC (902 publications) and the National Center for Immunization and Respiratory Diseases (521 publications) were the most productive (Figure 7). The most prolific journals were Journal of Medical Virology (432 publications) and PLOS ONE (195 publications) (Table 3).

**Figure 8:**
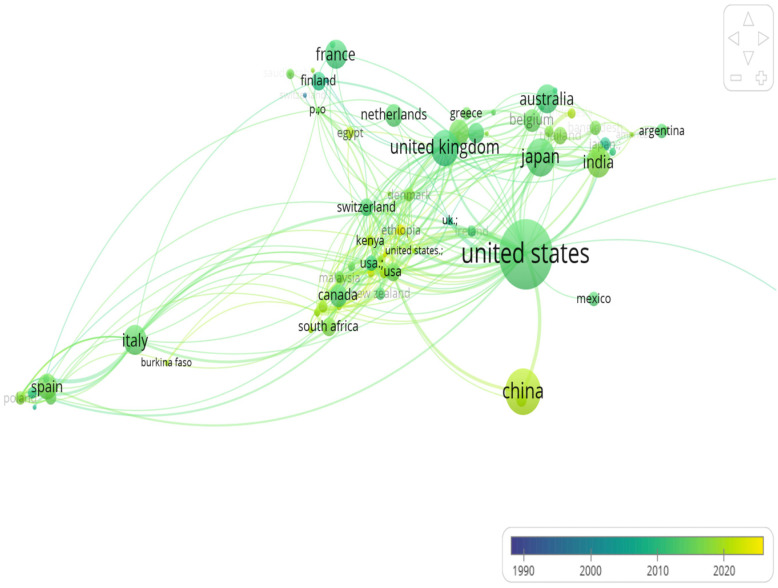
Global collaboration network (1990–2024). International co-authorship network (1990–2024). Nodes represent countries; the thickness of the links reflects the number of joint publications. Collaboration remains low overall, with <1% of total publications involving multiple countries.

Although international co-authorship publications represent less than 1% of total output, network mapping reveals a diverse collaborative structure: strong bilateral ties (notably U.S.–China), active hubs among Western countries (UK, Canada, Australia, various European countries), and meaningful links to Latin America, Asia, and parts of Africa (Figure 8). Thus, while the proportion of international collaborations is low, the collaboration network is structurally broad, integrating both North–South and South–South links.

### 3.3. A Research Landscape Dominated by Rotavirus

**Figure 9:**
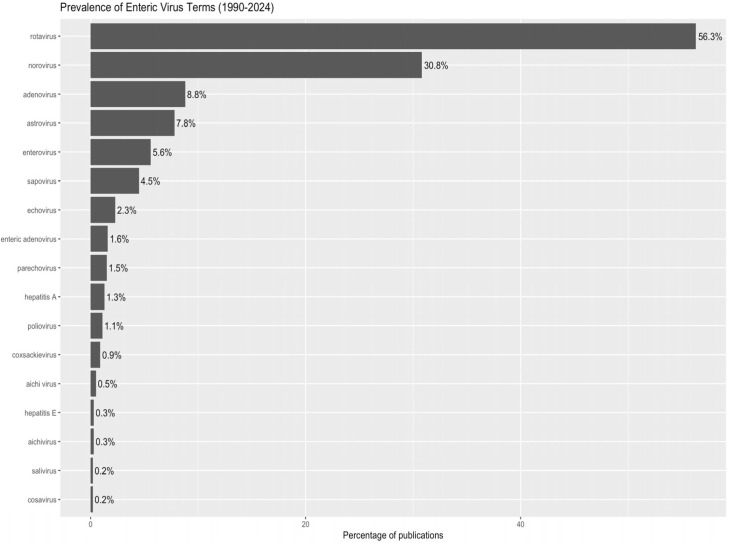
Prevalence of terms related to enteric viruses in the scientific literature (1990–2024). The percentage of publications including each virus in the title, keywords, or abstract is shown. The analysis revealed the thematic dominance of research on rotavirus and norovirus in the literature.

Text analysis of titles, abstracts, and keywords revealed that rotavirus was the predominant topic, appearing in 56.3% (*n* = 4543) of publications; norovirus came second, at 30.8% (*n* = 2491). Other enteric viruses (adenovirus, astrovirus, sapovirus, non-polio enterovirus, Aichi virus) each appeared in less than 13% of publications. These results indicate that overall bibliometric trends are primarily influenced by research on rotavirus (Figure 9). In other words, relational density exists, but the share of truly co-produced articles remains limited.

#### Comparison with General Biomedical Publication Trends

The increase in enteric publications between 2020 and 2021 reached +4.3% for the entire corpus. In comparison, the total volume of PubMed increased by +8.9% over the same period. When rotavirus-related articles are excluded, the enteric corpus shows a slight decrease (−0.7%). These sensitivity analyses confirm that the peak observed in 2021 is driven mainly by rotavirus and does not exceed the overall trend in biomedical publications during the COVID-19 pandemic.

This indicates that while the COVID-19 pandemic influenced research output, it did not disproportionately boost enteric virus research compared to the broader biomedical field, and the observed peak remains consistent with pre-existing trends in rotavirus research.

### 3.4. Trends in Epidemiology and Methods

Keyword trends indicate a concentration in pediatric infections and diarrheal disease: mentions of “children” remained consistently high (54.0% in 1990–1999 vs. 50.8% in 2020–2024), while “diarrhea” decreased from 56.9% to 41.1%. Pathogen-specific research shifted markedly: rotavirus vaccine research peaked during 2000–2019 (~19%), while norovirus research increased sharply (0% in the 1990s to 32.7% in 2020–2024). Astrovirus and adenovirus remained present at low frequencies (7–11%), while sapovirus gradually increased (0% → 6.0%). Methodological keywords such as RT-PCR rose from 4.7% to 15.0% before stabilizing at 10.4%, while genome sequencing expanded (10.6% → 26.2%). Mentions of wastewater also grew (0.9% → 2.5%), reflecting the rising importance of environmental surveillance (Table 4).

**Figure 10:**
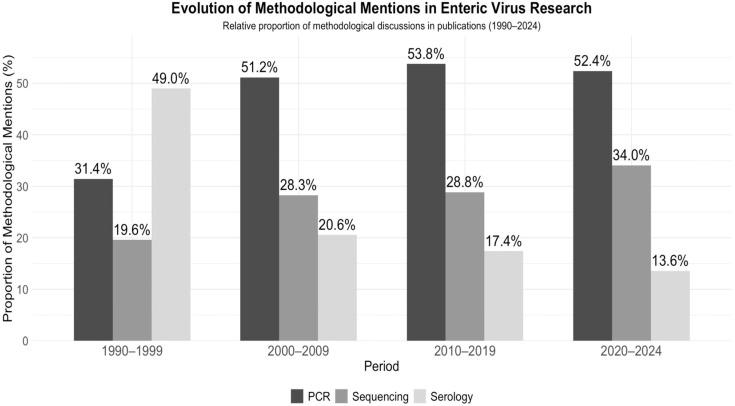
Evolution of Methodological Mentions (Data reflects methodological discussions in publication abstracts. PCR-based methods include RT-PCR and other PCR variants. Percentages represent relative proportion of methodological mentions within each period. When an article reported multiple diagnostic methods, each method was counted (full counting). Thus, percentages represent proportions of methodological mentions, not mutually exclusive study counts.) in Pathogenic Enteric Virus Research (1990–2024). Stacked bar chart depicting the frequency of diagnostic method mentions (RT-PCR, serology, and genome sequencing across four periods (1990s, 2000s, 2010s, and 2020–2024). The data are based on 1054 records from Scopus and PubMed. A single publication could contribute to multiple categories if it referenced more than one method. The *y*-axis shows the percentage of the total method mentions per time period accounted for by each method.

Methodological mentions evolved markedly: the presence of RT-PCR increased across decades, and mentions of genome sequencing rose to approximately 52.7% between 2020 and 2024. Genomic sequencing has become the dominant molecular approach, displacing serology in recent years (Figure 10).

**Figure 11:**
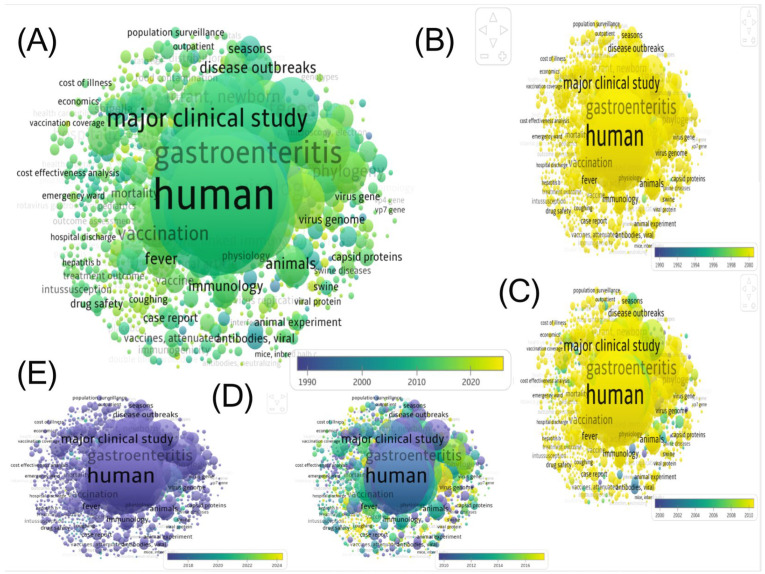
Chronological emergence of rotavirus-related keywords (1990–2024). (**A**): Temporal distribution of keywords, generated via VOSviewer. The color gradient (Purple → Yellow) reflects the chronological emergence of terms: purple represents dominant keywords during earlier periods (1990–2010), while yellow represents recent emerging keywords (post–2020). (**B**): 1990–2000: Focus on vaccinology (vaccine, intussusception follow-up), clinical burden (hospitalization, cost of illness, morbidity), and surveillance (population surveillance, age distribution). (**C**): 2000–2010: Emergence of advanced epidemiological studies (disease outbreaks, seasonality) and early pivot toward molecular diagnostic tools (genotype). (**D**): 2010–2017 (pre-COVID): Pivot toward molecular virology (molecular epidemiology, phylogeny, capsid proteins, vp7/vp4 gene) and basic research (animal experiments, antibodies, neutralizing). (**E**): 2018–2024 (post-COVID): Integration of genomics (virus genome), “One Health” approaches (wastewater surveillance, animals), and surveillance of specific populations (infection in pregnancy), reflecting the era of environmental surveillance and pandemic preparedness.

Co-occurrence mapping (Figure 11) reveals a marked chronological evolution of rotavirus research themes. This progression is visually apparent in the color gradient of Figure 11A (purple indicating older, and yellow indicating newer), and is detailed in the annotated panels B–E. The period from 1990 to 2010 was characterized by clinical and public health concerns, including “hospitalization,” “cost of illness,” and “intussusception.” A shift toward molecular tools (“genotype,” “molecular epidemiology,” “phylogeny,” “capsid proteins”) and animal models occurred between 2000 and 2017. Finally, the recent period (2018–2024) is characterized by the integration of viral genomics (“virus genome”), environmental surveillance, and “One Health” approaches (“wastewater surveillance,” “animals”), reflecting the field’s response to global pandemics and a growing focus on tracking zoonotic pathogens beyond traditional clinical settings.

### 3.5. Transmission Dynamics and Risk Factors

**Figure 12:**
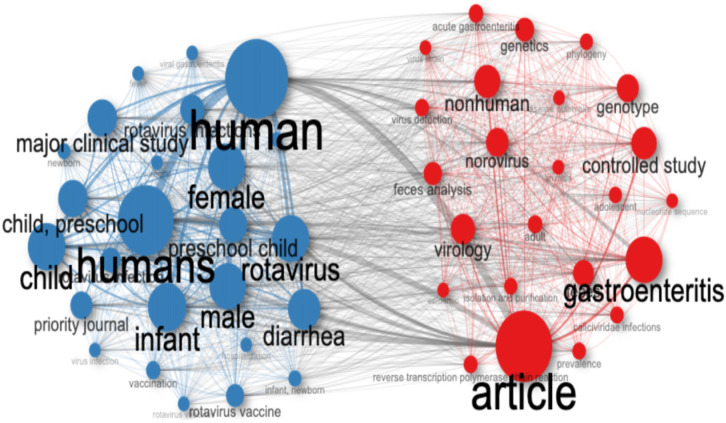
Co-occurrence network of transmission-related keywords (1990–2024). Convergence of terms such as ‘child,’ ‘diarrhea,’ ‘gastroenteritis,’ and environmental factors. This map highlights the intersection of clinical manifestations (‘diarrhea,’ ‘gastroenteritis’), vulnerable populations (‘child,’ ‘infant,’ ‘preschool child’), and transmission pathways that drive the spread of enteric viruses.

A transmission-keyword co-occurrence analysis revealed three primary transmission patterns: waterborne dissemination linked to environmental factors, outbreak propagation in communal settings, and transmission facilitated by inadequate sanitation infrastructure (Figure 12).

Our bibliometric analysis of 8320 studies published between 1990 and 2024 reveals that 6645 publications (80%) mention at least one risk factor. Among the 3311 rotavirus vaccine-related studies (Table 4), a subset of 2874 publications specifically mentioned risk factors and were included in the transmission dynamics analysis. The geographical distribution indicates modest coverage of regions of interest, with 211 studies (2.6%) focusing on West Africa and 298 studies (3.7%) on Southern Africa.

**Figure 13:**
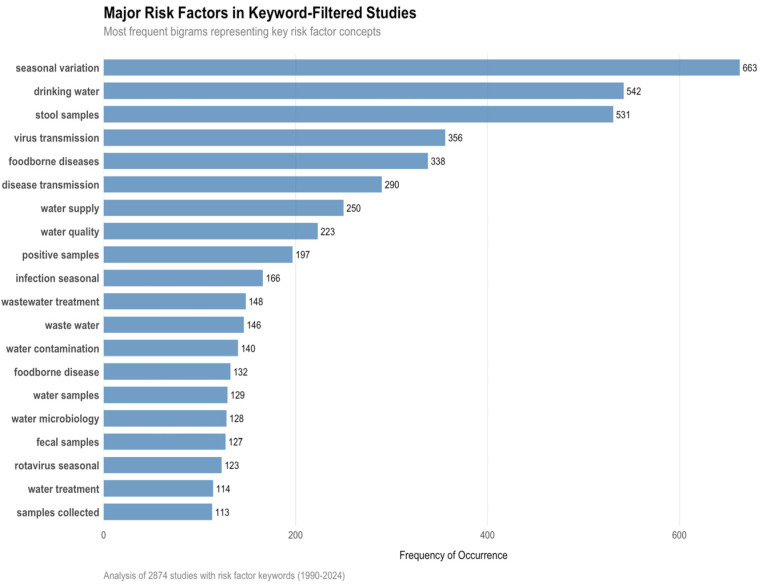
Frequency of principal transmission risk factors (*n* = 2874 studies). The visualization illustrates the geographical distribution of the 513 studies included in the regional risk factor analysis.

Thematic analysis identifies seasonal variation (12.9%) and sanitation and hygiene challenges (12.4%) as the main environmental risk factors reported in the literature. Access to safe drinking water (2.7%) and zoonotic transmission (3.0%) appear less frequently but remain significant concerns, while population density (0.4%) is rarely mentioned as a determining factor (Figure 13).

These results highlight the predominance of environmental and seasonal determinants in transmission dynamics, highlighting the importance of integrating these factors into disease prevention and control strategies.

### 3.6. Public Health Impact

As previously described, rotavirus research accounts for the most significant proportion of studies, comprising 56.3% of the corpus. Rotavirus vaccine research represents 39.8% of studies, followed by morbidity research (24.6%) and molecular characterization (22.7%). Studies on incidence and prevalence represent 12.5% of publications, while specific research on mortality remains rare (0.4%) (Table 5). The tangible impact of rotavirus vaccination is evident in the 50–60% reductions in hospitalizations documented in meta-analyses. Aggregated data have fundamentally informed global vaccination policies and implementation strategies worldwide.

## 4. Discussion

This systematic review (1990–2024) reveals a sustained growth in publications, with a compound annual growth rate (CAGR≈) of ~5.84%, peaking in 2021. There is also a notable geographical concentration, with five countries (the USA, China, Japan, India, and Brazil) accounting for ~92.4% of the literature. The corpus is dominated by rotavirus, whose vaccine impact is considerable: meta-analyses and global estimates report substantial reductions in pediatric hospitalizations and deaths after vaccine introduction.

Since ~2015–2020, there has been a rapid rise in genomic sequencing and the widespread adoption of Wastewater-based epidemiology (WBE) as tools for community monitoring and early detection—trends amplified by the COVID-19 response, which call for the construction of a sustainable global genomic surveillance network. However, real international collaboration and scientific production in high-burden regions remains limited (low transnational co-publications), which imposes clear political priorities: expanding vaccination coverage through support mechanisms (e.g., Gavi), strengthening local sequencing and WBE capacities, investing in WASH systems, and promoting data sharing and equitable financing to reduce inequalities in surveillance and response.

### 4.1. Rotavirus Predominance: Causes and Implications

The dominance of rotavirus in the literature is directly linked to its historical global burden. It has been the leading cause of severe, fatal diarrhea in infants and young children for decades. The development and deployment of rotavirus vaccines has created a virtuous cycle of research (efficacy assessment, strain surveillance, impact on morbidity/mortality) that has largely fueled scientific production.

Our sensitivity confirms that the observed increase in 2021 disappears when rotavirus studies are excluded, suggesting that the “2021 effect” is mainly linked to the specific dynamics of rotavirus research and the pandemic context. The early availability of robust, commercial diagnostic tests for rotavirus has also facilitated surveillance and epidemiological research, unlike other viruses such as norovirus or sapoviruses.

This disparity is not a methodological bias of our study, but rather a reflection of a real and profound bias in global health research priorities. Our bibliometric analysis thus becomes a meta-analysis of what the scientific community has chosen to study. However, this focus overlooks pathogens of increasing importance. Norovirus, for example, is a significant cause of gastroenteritis in all age groups and is now the leading cause of severe gastroenteritis in some countries where rotavirus vaccination is effective. The relative lack of research on non-polio enteroviruses and Aichi virus represents a critical gap, given their association with severe neurological disease and other systemic manifestations.

### 4.2. COVID-19 and the 2021 Publication Surge

The COVID-19 pandemic has significantly impacted research on enteric viruses. The WBE platforms developed for SARS-CoV-2 surveillance were rapidly adapted to monitor viruses such as norovirus, rotavirus, and hepatitis A/E, resulting in a significant increase in publications in 2021 [31,32]. Our data demonstrated this with a sharp increase in mentions of “wastewater” and genomic sequencing between 2020 and 2024.

This methodological boom has sped up the field, with analysis showing a 5.84% CAGR and a peak in 2021. However, output was geographically concentrated, mainly in the US, China, Japan, the UK, and India, with low levels of transnational co-authorship. This imbalance highlights an ongoing disparity between regions with robust methodological resources and those most severely affected by these viruses. The literature confirms that WBE is a powerful tool for enteric virus surveillance, particularly in areas with limited clinical systems [33,34]. However, translating this post-pandemic momentum into sustainable public health gains requires sustained investment, methodological standardization, and the transfer of technology to local laboratories. Without these, the increased interest risks being transitory [32,35]. Addressing geographic and collaborative disparities is essential. Our mapping highlights the need for targeted actions, such as fair funding, training, and the promotion of open data, to ensure local ownership of tools like WBE and genomics. This will help build sustainable, equitable surveillance systems that extend beyond COVID-19 [36,37].

### 4.3. Geographic Disparities

Our analysis reveals an apparent discrepancy between disease burden and scientific output: sub-Saharan Africa and Latin America account for only 7.6% of the global literature on pathogenic enteric viruses, despite experiencing some of the highest incidence and mortality rates worldwide. This underrepresentation aligns with previous studies highlighting persistent gaps in genomic surveillance and research funding in low- and middle-income countries [38]. Just five countries, primarily the United States, China, Japan, India, and Brazil, account for 92.4% of all publications, highlighting the imbalance between regions with high scientific capacity and those bearing the most significant disease burden.

Additionally, the impact of enteric infections is considerably greater in low- and middle-income countries (LMICs), yet scientific research remains focused in high-income countries (HICs). Recent estimates confirm that most DALYs and deaths due to enteric infections occur in regions with low socio-demographic indices, underscoring the gap between disease burden and scientific effort [39].

This asymmetry results in a lack of operational and contextual studies (WASH field assessments, local genomic surveillance, vaccine implementation studies) in the most affected regions, limiting the relevance and applicability of international recommendations. Recent analyses also reveal structural inequalities in investment and access to funding for health research, with a disproportionately small fraction of grant funding allocated to institutions based in low-resource countries [40,41].

The authorship inequalities and the so-called “parachute research” phenomenon, where external teams publish locally conducted work without sustained scientific leadership from local researchers, perpetuate these imbalances and reduce the operational impact of research in the field. Our analysis reveals that these geographic disparities extend beyond the sheer volume of publications to the ownership of research itself. The stark difference between a country’s total production and its corresponding authorship rate uncovers a pervasive power imbalance. The high corresponding authorship rates in countries like Japan (~20%) and the UK (~18%) signify robust local leadership. Conversely, the very low rates in countries like Mozambique (3.2%) and Nigeria (6.5%) are characteristic of ‘parachute research,’ where researchers from high-income countries lead studies in low-income settings with minimal local scientific leadership [42]. This practice, while generating data, often hinders sustainable local capacity building and can result in research agendas that are misaligned with regional priorities.

Critical reviews document that, for many studies conducted in Africa and other developing regions, less than half of the articles feature local authors in the first or last position, illustrating the need to invest in equitable partnerships and local capacity [42]. International collaborations remain limited, with less than 1% of articles involving at least one transnational collaboration. Authorship asymmetries further reflect systemic inequalities: in Africa, local researchers held the first author position in only 49.8% of studies and the last author position in 41.3%, illustrating the persistence of the “parachute research” dynamic that hinders contextual relevance and sustainable capacity building.

These authorship inequalities are both a cause and an effect of the funding imbalances noted previously. When grants are awarded to institutions in high-income countries, leadership typically remains in place. This creates a vicious cycle that limits the ability of researchers in high-burden regions to compete for future funding and establish independent research programs.

Funding inequalities exacerbate these trends, as less than 10% of global investments in health research are directed to low- and middle-income countries, despite these countries accounting for approximately 90% of preventable morbidity and mortality [43,44]. Limited laboratory capacity, high publication fees, and restricted access to high-impact journals further limit equity of participation. By quantitatively mapping these disparities across 35 years of research, our study provides robust evidence to guide targeted investments in sequencing networks, training programs, and long-term North–South partnerships, thereby ensuring that research agendas more accurately reflect the global burden of disease and public health needs. Therefore, closing the equity gap requires a conscious shift towards genuine co-creation, where partnerships are designed from the outset to build leadership, capacity, and ownership within the most affected communities.

### 4.4. Methodological Trends

Our analysis demonstrates a gradual methodological transition in enteric virus research. While serological tests were dominant in the 1990s, their use has steadily declined over the past three decades, now accounting for less than 15% of methodological mentions. This reflects global diagnostic trends, where molecular techniques are increasingly supplanting serology due to their superior sensitivity and specificity in detecting acute infections [45]. PCR rapidly established itself as the cornerstone of enteric virus diagnosis from the 2000s onwards and remains the most widely used method. This stability is consistent with previous bibliometric and clinical reviews that have identified RT-PCR as the gold standard for norovirus and rotavirus surveillance [46,47].

Meanwhile, sequencing approaches, including next-generation sequencing (NGS), have experienced significant growth since 2015, now accounting for nearly a third of methodological citations in the most recent period. This expansion follows international studies that document the use of whole-genome sequencing for epidemic reconstruction and mapping the cross-border transmission of foodborne viruses [48]. Beyond epidemic detection, metagenomic applications have opened up new possibilities for pathogen discovery and the identification of zoonotic spillover, highlighting the broader relevance of genomics for One Health programs [49].

Despite these advances, our mapping confirms that genomic datasets and vaccine efficacy studies remain heavily concentrated in high-income countries. This geographic imbalance has also been noted in recent reviews on rotavirus and norovirus, which highlight that the underrepresentation of African and Latin American strains limits the accuracy of molecular surveillance and delays the detection of emerging variants [50]. These limitations are particularly concerning, given that rotavirus still accounts for 28–33% of pediatric diarrhea hospitalizations worldwide. While high-income countries have achieved over 80% reductions in hospitalizations following vaccine introduction, comparable benefits have remained difficult to achieve in low-resource settings [51]. New vaccine developments, including animal–human polyvalent and reassortant candidates, highlight future opportunities but also reinforce the urgent need to expand genomic and vaccine research capacity in underrepresented regions [52].

### 4.5. Transmission Dynamics and Risk Factors

Our analysis of 2874 studies explicitly addressing risk factors for enteric virus transmission highlights the predominance of environmental and infrastructural determinants. Bigram analysis revealed that water and sanitation factors were the predominant concern (8.4% of risk factor studies), followed by hygiene practices (2.7%). High urban population density was a less common factor (0.4%).

Examination of the regional distribution of these factors revealed distinct trends: seasonal variations were documented in 22.3% of studies from West Africa, compared to 16.8% in those from Southern Africa, representing significant regional disparity. This contrast underscores the need for locally tailored surveillance strategies that reflect regional ecological contexts and climatic patterns.

Zoonotic transmission was mentioned in 2.1% of studies overall, with a slightly higher reporting rate in Southern Africa (2.2%) than in West Africa (2.0%), suggesting different epidemiological interfaces with livestock and wildlife across regions. The geographical distribution of the regional literature revealed a higher representation of research in Southern Africa (309 studies, 60.2%) compared to West Africa (204 studies, 39.8%), which may influence the characterization of risk factors in the scientific data.

Co-occurrence network analysis revealed a strong clustering of the terms “infant,” “diarrhea,” and “sanitation,” illustrating the intersection of clinical, environmental, and social determinants of health.

These findings highlight the importance of WASH interventions, which cover 11.1% of water, sanitation, and hygiene factors. They also underscore the importance of incorporating climate variables and zoonotic pathways into models. Combining WASH, vaccination, hygiene education, and environmental surveillance is crucial for sustainably reducing pediatric morbidity and mortality in areas with high prevalence.

Climate change is increasingly affecting the environmental factors of enteric virus transmission. Changes in rainfall and water quality can lead to drinking water contamination and post-flood outbreaks of various pathogens. These are linked to higher risks of emergence and spread, underscoring the need to incorporate meteorological data into transmission models [53,54].

Wastewater-based epidemiology (WBE) is emerging as a valuable tool for early detection of viral circulation and monitoring trends, including those affected by climate events. Combining WBE data, weather, and sociodemographic indicators can enhance the accuracy of early warning systems’ predictions [53]. Few studies integrate climate variables and microbiological models at the national level, missing a key chance to improve health preparedness and target WASH and vaccination efforts [55].

### 4.6. Thematic Focus at the Expense of Antivirals

Beyond established preventive strategies such as rotavirus vaccination, WASH interventions, and outbreak control, antiviral therapies for enteric viruses remain limited [56]. No specific drugs have yet been approved for routine use, and treatment focuses primarily on symptom relief. Some agents, including pleconaril [57], nitazoxanide [58,59], favipiravir, and ribavirin, have been tested with variable or inconclusive results [60]. Meanwhile, emerging options such as monoclonal antibodies, polymerase inhibitors, and small-molecule antivirals are still in the preclinical or early clinical evaluation phases [61,62,63].

Our bibliometric analysis aimed to map the overall research landscape, encompassing not only the volume of publications but also their broader context. A deeper exploration of the corpus, prompted by the initial results, uncovered a significant thematic imbalance in research priorities. While the broad theme of “treatment” is indeed essential, accounting for 17.6% of the corpus (*n* = 1608/8320), a detailed textual analysis using specific terms (such as antivirals like nitazoxanide, pleconaril, monoclonal antibodies, polymerase inhibitors) shows a different picture: research focused explicitly on the development and assessment of targeted antiviral therapies is a small niche, making up less than 1.5% of the literature.

This discrepancy is due to the nature of the publications included in the broad search. Analysis of the most common keywords in this subset, rotavirus (*n* = 228), humans (*n* = 224), norovirus (*n* = 157), diarrhea (*n* = 125), gastroenteritis (*n* = 115), infant (*n* = 114), shows that these are mostly observational clinical studies, trials of supportive treatments (such as probiotics, *n* = 42), or general reviews mentioning “treatment” in a broad sense, rather than preclinical or clinical studies of specific antiviral molecules.

This bibliometric gap in antiviral research highlights broader issues: the challenge of developing broad-spectrum compounds for picornaviruses and caliciviruses, the limited incentives for diseases in low- and middle-income countries, and a historical focus on vaccine development. The low antiviral focus signifies an actual gap in therapeutic innovation, not a methodological flaw. It supports our main finding: enterovirus research focuses on a few strategies, such as surveillance and vaccination, neglecting other vital areas for public health, particularly for immunocompromised patients and severe epidemics where antivirals are crucial. Closing this gap requires targeted funding and public–private partnerships to foster research in this neglected area.

### 4.7. Policy Implications

Quantified regional disparities in research focus and risk factors highlight the need to move beyond one-size-fits-all models. Our findings provide a clear mandate for evidence-based, context-specific strategies. To close the knowledge and equity gaps identified in this study, we recommend the following policy directions:

- Expand equitable rotavirus immunization coverage, particularly in low- and middle-income countries (LMICs), through Gavi-supported platforms and reinforcement of cold-chain logistics;
- Scale genomic surveillance infrastructures in underserved regions by funding local laboratories, training scientists, and ensuring access to sequencing tools and data analysis platforms;
- Adopt integrated One Health surveillance in zoonotic hotspots by combining human, animal, and environmental monitoring to improve early detection and outbreak response;
- Invest in sustainable WASH (Water, Sanitation, and Hygiene) systems, recognizing that diagnostics and vaccines are insufficient without foundational public health infrastructure;
- Ensure inclusive knowledge sharing and data equity by strengthening open-access repositories, FAIR data standards, and inclusive platforms for LMIC researchers (e.g., preprints, community peer review);
- Incorporate climate and socio-demographic data into future surveillance models to anticipate outbreak risks in vulnerable populations and improve public health preparedness.
- Address gender and equity barriers in access to prevention, diagnosis, and care, with a focus on context-specific health education and resource allocation;
- Together, these measures provide a roadmap for stakeholders to strengthen surveillance, prevention, and research equity, thereby reducing the global burden of enteric viruses.

## 5. Conclusions and Limitations

This first comprehensive bibliometric analysis of 35 years (1990–2024) of research on pathogenic enteric viruses documents a clear methodological shift from serology to routine molecular diagnostics and then to genomic and environmental surveillance, with an acceleration of wastewater-based epidemiology during and after the COVID-19 pandemic, while simultaneously revealing persistent and deep geographic inequalities in research capacity, funding, and infrastructure. While recent methodological advances create robust opportunities for earlier detection, more accurate genotyping, and environmental monitoring, these benefits remain unevenly distributed: scientific output and genomic capacity are concentrated in a small number of high-income countries. In contrast, many high-burden regions remain underrepresented, and parachute research practices limit sustainable local capacity building.

These findings should be interpreted in the context of several significant limitations. Although English is the primary language of scientific communication, we recognize that our analysis, limited to publications in English and French, could underestimate the contribution of research produced in other languages, notably Spanish or Portuguese, which are often published in regional journals; Second, manuscripts available only on preprint servers at the time of data entry were not included, creating a potential lag in tracking the latest trends; Third, bibliometric indicators such as publication and citation counts measure quantity and visibility but do not directly assess methodological rigor, reproducibility, or clinical relevance. The exclusion of the 108 records without affiliation data represents a minor limitation of our analysis, as it prevented quantification of the contribution of these publications to research trends by country or region. Finally, although we combined automated classification with manual validation, some misclassification of topics or areas is inevitable in large-scale text analyses. Despite these constraints, the evidence supports an urgent, equity-focused agenda: democratizing access to genomic and environmental surveillance tools, prioritizing targeted investments in local laboratories and WASH infrastructure, and fostering genuine, co-produced partnerships that involve researchers from low- and middle-income settings. Aligning funding and research priorities with this roadmap will be essential to transforming technical advances into equitable and sustainable gains in public health worldwide.

Despite these constraints, our findings provide unequivocal evidence in support of an urgent, equity-focused agenda. To translate technical advances into equitable and sustainable gains in global public health, we propose a roadmap built on three pillars: (1) Democratizing tools by expanding access to genomic and environmental surveillance technologies; (2) Prioritizing targeted investments in local laboratory capacity and WASH infrastructure; and (3) Fostering genuine co-produced partnerships that center and engage researchers from low- and middle-income settings. Aligning global funding and research priorities with this vision is no longer just an ethical imperative but a strategic necessity for effective pandemic preparedness and health security worldwide.

## Figures and Tables

**Table 1:** Epidemiological, transmission, and prevention characteristics of the main pathogenic enteric viruses.

Family	Virus	Key Epidemiological Features	Transmission Routes	Public Health Impact	Prevention/Treatment	Research Challenges	References
Sedoreoviridae	Rotavirus	Leading cause of severe pediatric diarrhea globally; peak incidence in children <5 years	Fecal–oral, contaminated water/food	High morbidity and mortality in LMICs; seasonal epidemics	Vaccination, WASH interventions, supportive care	Strain diversity, vaccine escape variants, limited surveillance in LMICs	[3]
Caliciviridae	Norovirus, Sapovirus	Major cause of gastroenteritis across all ages; frequent outbreaks in closed settings	Fecal–oral, contaminated food/water, fomites	High burden in both HICs and LMICs; outbreaks in hospitals, cruise ships, schools	Hygiene, outbreak control measures, vaccine development ongoing	High genetic diversity, short-lived immunity, limited vaccines	[16,17]
Adenoviridae	Enteric adenovirus (F40/F41)	Causes diarrhea primarily in infants and young children	Fecal–oral	Moderate global burden; associated with sporadic outbreaks	Supportive care, hygiene measures	Limited epidemiological data, few diagnostics in LMICs	[18,19]
Astroviridae	Astrovirus	Often mild diarrhea in children; some severe cases in immunocompromised	Fecal–oral	Underrecognized; global incidence unclear	Hygiene, supportive care	Limited surveillance, low research attention	[14,20]
Picornaviridae ^1^	Enteroviruses (genus Enterovirus; e.g., coxsackievirus, echovirus). Other enteric picornaviruses include human parechoviruses (HPeV; genus Parechovirus) and Aichi virus (AiV; genus Kobuvirus)	Clinical spectrum ranges from gastroenteritis to occasional systemic/CNS disease; some types associated with severe neonatal disease (e.g., certain HPeV types)	Fecal–oral, waterborne	Significant pediatric morbidity in outbreaks; variable detection across settings	Supportive care; some experimental antivirals	Diagnostic heterogeneity, limited surveillance, taxonomic and nomenclature confusion in older literature	[21,22,23,24]
Hepeviridae	Hepatitis E virus (HEV)	Enterically transmitted hepatitis; high risk in pregnant women	Fecal–oral, contaminated water	Severe outbreaks in LMICs; high mortality in pregnancy	Vaccination (restricted availability), WASH	Poor surveillance, limited vaccine coverage outside China	[25]
Hepatoviridae	Hepatitis A virus (HAV)	Self-limiting acute hepatitis; widespread global exposure	Fecal–oral	Moderate burden; epidemics in regions with poor sanitation	Vaccination, WASH	Limited genomic surveillance, outbreak prediction challenges	[26]

LMICs: Low- and middle-income countries. ^1^ Non-polio enteroviruses (NPE) are members of the genus Enterovirus (family Picornaviridae) and include species such as coxsackieviruses and echoviruses. Human parechoviruses (HPeV) are classified in the separate genus Parechovirus (family Picornaviridae), and Aichi virus (AiV) belongs to the genus Kobuvirus (family Picornaviridae). We therefore treat NPE (Enterovirus genus) separately from other enteric picornaviruses (HPeV, AiV) in the manuscript and analyses.

**Table 2:** Research Leadership and Participation of the Top 10 Most Productive Countries (1990–2024).

Country	Total Publications	Corresponding Authorships	Leadership Share (%) *
USA	2205	349	15.8
China	932	80	8.6
Japan	641	129	20.1
India	438	64	14.6
Brazil	363	45	12.4
Italy	335	48	14.3
France	325	70	21.5
South Korea	309	38	12.3
Australia	282	69	24.5
Germany	223	24	10.8

* Leadership Share was calculated as (Corresponding Authorships/Total Publications) × 100. A higher percentage indicates a greater proportion of studies where researchers from that country held the senior leadership position.

**Table 3:** Top 10 Publishing Countries, Institutions, and Journals (1990–2024).

Country	Articles	Institution	Articles	Journal	Articles
USA	3180	Centers for Disease Control and Prevention	902	Journal of Medical Virology	432
China	1912	National Center for Immunization and Respiratory Diseases	521	Vaccine	378
Japan	1034	University of Liverpool	362	Journal of Clinical Microbiology	246
India	874	Christian Medical College	341	Pediatric Infectious Disease Journal	214
Brazil	689	Baylor College of Medicine	318	PLOS One	195
Italy	602	International Centre for Diarrhoeal Disease Research	287	Viruses	168
UK	511	Emory University	263	Epidemiology and Infection	151
Spain	498	University of Virginia	244	Archives of Virology	147
France	472	Chiang Mai University	229	Journal of Infectious Diseases	139
South Korea	438	Nihon University School of Medicine	196	BMC Infectious Diseases	133

Summary of the top 10 leading contributors to enteric-virus research based on publication count.

**Table 4:** Decadal evolution of selected author keywords in pathogenic enteric viruses research (1990–2024).

Keyword	1990–1999 %	2000–2009 %	2010–2019 %	2020–2024 %
Children	54.0	48.5	51.7	50.8
Gastroenteritis	42.3	52.5	55.1	46.0
Diarrhea	56.9	44.7	42.0	41.1
Norovirus	0.0	26.6	35.9	32.7
Genome sequencing	10.6	16.5	19.4	26.2
Gastroenteritis	42.3	52.5	55.1	46.0
Rotavirus vaccines	18.6	17.0	19.2	16.7
Outbreak	10.8	24.3	18.0	15.6
RT-PCR	4.7	15.0	13.8	10.4
Adenovirus	9.5	7.4	7.1	9.8
Astrovirus	11.1	9.5	6.7	7.8
Sapovirus	0.0	3.5	4.7	6.0
Molecular epidemiology	2.7	6.1	5.8	4.9
Wastewater	0.9	1.8	1.9	2.5
Serotyping	26.6	9.9	3.6	1.8
RT-PCR	4.7	15.0	13.8	10.4

Percentages represent the proportion of publications containing the keyword in each decade. A single publication could contain multiple keywords.

**Table 5:** Descriptive Analysis of Rotavirus Research Indicators.

Study Type	Number of Studies	Percentage
Vaccine	3311	39.8%
Morbidity	2045	24.6%
Molecular/Genetic	1890	22.7%
Incidence/Prevalence	1042	12.5%
Mortality	31	0.4%
Other	1	0.0%

Statistical summary for incidence, mortality, and morbidity extracted from included articles. Indicates high variability and skewness in reported values.

## Data Availability

All data generated and analyzed during this study were included in this published article, and any further datasets are available from the corresponding author upon reasonable request.

## Supplementary Materials

The following supporting information can be downloaded at: https://www.mdpi.com/article/10.3390/pathogens14090938/s1, File S1: Search strategy of Enteric viruses.
