# Supplementary material for: Global Research Trends on Major Pathogenic Enteric Viruses (1990–2024): A Bibliometric Analysis of Epidemiology, Transmission, and Public Health Impact
Source: Pathogens. 2025 Sep 16;14(9):938. doi: 10.3390/pathogens14090938 (PMC12472404; doi:10.3390/pathogens14090938)
Supplement: Supplementary file 1 [file pathogens-14-00938-s001.zip › pathogens-3825476-supplementary.pdf]

1. Search Queries:

- **Scopus Query:**

TITLE-ABS-KEY (
("rotavirus" OR "norovirus" OR "enteric adenovirus" OR "astrovirus" OR "sapovirus" OR "enterovirus" OR "coxsackievirus" OR "coxsackievirus A" OR "coxsackievirus B" OR "echovirus" OR
"Aichi virus" OR "parechovirus" OR "picornaviridae")
AND
("viral gastroenter*" OR "gastroenteritis" OR "diarrh*" OR "vomit*" OR "dehydrat*" OR "fecal-oral transmission" OR "waterborne diseases" OR "foodborne diseases")
AND
("epidemiol*" OR "transmiss*" OR "diagnos*" OR "molecular surveillance" OR "genomic sequencing" OR
"prevent*" OR "vaccinat*" OR "antiviral therap*" OR "WASH" OR "sanitation" OR "climate change")
)
AND (LIMIT-TO (DOCTYPE , "ar") OR LIMIT-TO (DOCTYPE , "re"))
AND (EXCLUDE (SUBJAREA , "VETE") OR EXCLUDE (SUBJAREA , "AGRI"))
AND (EXCLUDE (PUBYEAR , 2025))
AND (LIMIT-TO (LANGUAGE , "English") OR LIMIT-TO (LANGUAGE , "French")).

- **PubMed Query:**

(
(
"rotavirus"[Title/Abstract] OR "norovirus"[Title/Abstract] OR "enteric adenovirus"[Title/Abstract] OR
"astrovirus"[Title/Abstract] OR "sapovirus"[Title/Abstract] OR "enterovi-rus"[Title/Abstract] OR
"coxsackievirus"[Title/Abstract] OR "echovirus"[Title/Abstract] OR "Aichi virus"[Title/Abstract] OR
"parechovirus"[Title/Abstract] OR "picornaviridae"[Title/Abstract]
)
AND
(
"viral gastroenteritis"[Title/Abstract] OR "gastroenteritis"[Mesh] OR "diar-rhea"[Mesh] OR
"vomiting"[Mesh] OR "dehydration"[Mesh] OR "fecal-oral route"[Mesh] OR
"waterborne diseases"[Mesh] OR "foodborne diseases"[Mesh]
)
AND
(
"epidemiology"[Mesh] OR "disease transmission"[Mesh] OR "diagnosis"[Mesh] OR
"molecular surveillance"[Title/Abstract] OR "genomic sequenc-ing"[Title/Abstract] OR
"prevention"[Mesh] OR "vaccination"[Mesh] OR "antiviral agents"[Mesh] OR
"sanitation"[Mesh] OR "climate change"[Mesh]
)
)
AND (english[Language] OR french[Language])
AND ("1990/01/01"[Date - Publication] : "2024/12/31"[Date - Publication])

AND (humans[Mesh]).

PubMed: 1572 results (pubmed_file.txt)

Scopus: 8445 results (Scopus_file.csv)

2. Script to import files into R

library(bibliometrix)
PubMed <- convert2df("/Users/mac/Downloads/Nouveau dossier contenant des éléments/pubmed_file.txt", dbsource = "pubmed", format = "pubmed")
Scopus <- convert2df("/Users/mac/Downloads/scopus_file.csv", dbsource = "scopus", format = "csv")
Database <- mergeDbSources(PubMed, Scopus, remove.duplicated = TRUE)
Database "9137" avec 880 duplicated documents have been removed
install.packages("openxlsx")
library(openxlsx)
write.xlsx(Database, file = "/Users/mac/Downloads/Database.xlsx", rowNames = FALSE)

```
install.packages("dplyr")
install.packages("tidyverse")
library(dplyr)
id_col <- "ID"
library(dplyr)
df_filtered <- df %>%
  filter(!is.na(PY) & PY >= 1990 & PY <= 2024) %>%
  distinct(.data[[id_col]], .keep_all = TRUE)
```

3. west africa, ouest Africa, and risk factor

```
library(dplyr)
library(stringr)
library(openxlsx)
df_filtered <- df %>%
  filter(!is.na(PY) & PY >= 1990 & PY <= 2024) %>%
  distinct(ID, .keep_all = TRUE)
cat("Analysis restricted to 1990-2024 period:", nrow(df_filtered), "documents\n")
if(!"all_text" %in% colnames(df_filtered)) {
  text_columns <- c("title_text", "abstract_text", "keywords_text", "affil_text")
  available_text_cols <- text_columns[text_columns %in% colnames(df_filtered)]
  if(length(available_text_cols) > 0) {
    df_filtered$all_text <- apply(df_filtered[, available_text_cols], 1,
                              function(x) paste(na.omit(x), collapse = " "))
    cat("Column 'all_text' created from:", paste(available_text_cols, collapse=", "), "\n")
```

```
  } else {
    stop("No text columns available for 'all_text'. Cannot continue.")
  }
}

west_africa <- c("ghana","nigeria","senegal","cote d'ivoire","côte d'ivoire","mali","burkina faso","benin","togo")
southern_africa <- c("botswana","namibia","south africa","zimbabwe","zambia","malawi")
pattern_west <- paste0("\\b(", paste0(west_africa, collapse="|"), ")\\b")
pattern_southern <- paste0("\\b(", paste0(southern_africa, collapse="|"), ")\\b")
if(!"in_west_africa" %in% colnames(df_filtered)) {
  df_filtered <- df_filtered %>%
    mutate(in_west_africa = str_detect(all_text, regex(pattern_west, ignore_case = TRUE)))
}
if(!"in_southern_africa" %in% colnames(df_filtered)) {
  df_filtered <- df_filtered %>%
    mutate(in_southern_africa = str_detect(all_text, regex(pattern_southern, ignore_case = TRUE)))
}

risk_patterns <- c(
  "risk factor", "determinant",
  "age", "gender", "sex", "socioeconomic",
  "diabetes", "hypertension", "hiv", "aids",
  "tuberculosis", "obesity", "malnutrition",
  "smoking", "alcohol", "drug use",
  "poverty", "education", "income",
  "sanitation", "hygiene", "water access", "clean water", "drinking water",
  "population density", "overcrowding",
  "seasonal", "rainy season",
  "zoonotic", "animal transmission"
)
risk_pattern_combined <- paste(risk_patterns, collapse="|")
df_filtered <- df_filtered %>%
  mutate(risk_detected = str_detect(all_text, regex(risk_pattern_combined, ignore_case = TRUE)))
risk_total <- sum(df_filtered$risk_detected, na.rm = TRUE)
cat("Number of documents with detected risk factors (1990-2024):", risk_total, "(", round(risk_total/nrow(df_filtered)*100,1), "%)\n")

west_africa_count <- sum(df_filtered$in_west_africa, na.rm=TRUE)
```

```
southern_africa_count <- sum(df_filtered$in_southern_africa, na.rm=TRUE)
west_percent <- round(west_africa_count / nrow(df_filtered) * 100, 1)
southern_percent <- round(southern_africa_count / nrow(df_filtered) * 100, 1)
if(risk_total > 0) {
  risk_details <- data.frame(
    sanitation = round(sum(str_detect(df_filtered$all_text[df_filtered$risk_detected],
regex("sanitation|hygiene", ignore_case=TRUE))) / risk_total * 100,1),
    water_access =
round(sum(str_detect(df_filtered$all_text[df_filtered$risk_detected], regex("water
access|clean water|drinking water", ignore_case=TRUE))) / risk_total * 100,1),
    population_density =
round(sum(str_detect(df_filtered$all_text[df_filtered$risk_detected],
regex("population density|overcrowding", ignore_case=TRUE))) / risk_total * 100,1),
    seasonal = round(sum(str_detect(df_filtered$all_text[df_filtered$risk_detected],
regex("seasonal|rainy season", ignore_case=TRUE))) / risk_total * 100,1),
    zoonotic = round(sum(str_detect(df_filtered$all_text[df_filtered$risk_detected],
regex("zoonotic|animal transmission", ignore_case=TRUE))) / risk_total * 100,1)
  )
}
risk_results <- data.frame(
  Total_studies = nrow(df_filtered),
  Risk_studies = risk_total,
  Risk_percentage = round(risk_total/nrow(df_filtered)*100,1),
  West_Africa_studies = west_africa_count,
  West_Africa_percent = west_percent,
  Southern_Africa_studies = southern_africa_count,
  Southern_Africa_percent = southern_percent,
  Sanitation_percent = risk_details$sanitation,
  Water_percent = risk_details$water_access,
  Population_density_percent = risk_details$population_density,
  Seasonal_percent = risk_details$seasonal,
  Zoonotic_percent = risk_details$zoonotic
)
write.xlsx(risk_results, file="/Users/mac/Downloads/RiskFactor_1990_2024.xlsx",
rowNames=FALSE)
cat("Risk factor results exported to Excel.\n")
```

4. **Then I proceeded with the biblioshiny analyses, starting by applying a filter to select only the period between 1990 and 2024.**

```
c("ggplot2", "dplyr", "maps", "viridis", "sf", "rnaturalearth", "rnaturalearthdata", "scales"))

library(ggplot2)
library(dplyr)
library(sf)
library(rnaturalearth)
library(rnaturalearthdata)
library(scales)

world <- ne_countries(scale = "medium", returnclass = "sf")
world <- world %>%
  mutate(country_name = case_when(
    name == "United States of America" ~ "United States",
    name == "United Kingdom" ~ "United Kingdom",
    name == "Dem. Rep. Congo" ~ "Democratic Republic of the Congo",
    name == "Congo" ~ "Republic of Congo",
    TRUE ~ name
  ))
extract_countries <- function(text) {
  countries <- c("USA", "United States", "China", "UK", "United Kingdom", "Japan", "Germany",
                 "France", "Canada", "Australia", "South Africa", "Nigeria", "Brazil", "India",
                 "Italy", "Spain", "Netherlands", "Ghana", "Senegal", "Mali", "Burkina Faso",
                 "Benin", "Togo", "Botswana", "Namibia", "Zimbabwe", "Zambia", "Malawi",
                 "Russia", "South Korea", "Mexico", "Argentina", "Chile", "Colombia", "Peru",
                 "Egypt", "Kenya", "Ethiopia", "Uganda", "Tanzania", "Pakistan", "Bangladesh",
                 "Indonesia", "Thailand", "Vietnam", "Philippines", "Malaysia", "Turkey",
                 "Saudi Arabia", "Iran", "Israel", "Sweden", "Norway", "Denmark", "Finland",
                 "Switzerland", "Austria", "Belgium", "Portugal", "Greece", "Poland")

  text_lower <- tolower(text)
  countries_lower <- tolower(countries)
  detected_countries <- countries[sapply(countries_lower, function(x) grepl(x, text_lower, fixed = TRUE))]
  if (length(detected_countries) > 0) {
    return(detected_countries[1])
  } else {
    return(NA)
  }
}
if(!"country" %in% colnames(df_filtered)) {
  df_filtered$country <- sapply(df_filtered$all_text, extract_countries)
}
```

```
df_filtered <- df_filtered %>%
  mutate(country = case_when(
    country %in% c("USA", "United States") ~ "United States",
    country %in% c("UK", "United Kingdom") ~ "United Kingdom",
    TRUE ~ country
  ))
publications_by_country <- df_filtered %>%
  filter(!is.na(country)) %>%
  count(country, name = "publications") %>%
  arrange(desc(publications))
world_data <- world %>%
  left_join(publications_by_country, by = c("country_name" = "country"))
sequential_palette <- c("#FFF7BC", "#FEC44F", "#D95F0E", "#A63603", "#7F0000")
p <- ggplot(data = world_data) +
  geom_sf(aes(fill = publications), color = "white", size = 0.1) +
  scale_fill_gradientn(
    colours = sequential_palette,
    name = "Publications",
    trans = "pseudo_log",
    na.value = "grey90",
    labels = comma_format()
  ) +
  labs(
    title = "Global Distribution of Enteric Virus Publications (1990-2024)",
    subtitle = "Color intensity corresponds to publication volume. Grey indicates no data.",
    caption = "Data: Scopus and PubMed | Sub-Saharan Africa remains underrepresented despite
high disease burden"
  ) +
  theme_void() +
  theme(
    plot.title = element_text(hjust = 0.5, face = "bold", size = 16),
    plot.subtitle = element_text(hjust = 0.5, size = 12, margin = margin(b = 10)),
    plot.caption = element_text(hjust = 0.5, size = 10, color = "gray50"),
    legend.position = "right"
  )

top_5_countries <- head(publications_by_country, 5)
inset_plot <- ggplot(top_5_countries, aes(x = reorder(country, publications), y = publications,
fill = publications)) +
  geom_col() +
  scale_fill_gradientn(colours = sequential_palette, guide = "none") +
  geom_text(aes(label = comma(publications)), hjust = -0.1, size = 3) +
  coord_flip() +
  labs(x = NULL, y = NULL, title = "Top 5 Countries") +
  theme_minimal(base_size = 9) +
  theme(
    axis.text.x = element_blank(),
    panel.grid.major = element_blank(),
    plot.title = element_text(face = "bold", size = 10),
```

```
    plot.background = element_rect(fill = "white", color = NA)
  )

p_final <- p +
  annotation_custom(
    grob = ggplotGrob(inset_plot),
    xmin = -170, xmax = -100,
    ymin = -50, ymax = -10
  )
print(p_final)
ggsave("/Users/mac/Downloads/global_enteric_virus_map_corrected.png",
       p_final, width = 14, height = 8, dpi = 300, bg = "white")
```

5. Generation of Figure 9 showing the prevalence of enteric viruses

```
library(ggplot2)
library(dplyr)
library(scales)
library(stringr)

enteric_viruses <- c(
  "rotavirus", "norovirus", "astrovirus", "sapovirus", "adenovirus",
  "enterovirus", "poliovirus", "coxsackievirus", "echovirus", "hepatitis A",
  "hepatitis E", "aichivirus", "cosavirus", "salivirus", "parechovirus"
)
calculate_virus_prevalence <- function(data, virus_list) {
  total_publications <- nrow(data)

  prevalence <- data.frame(
    virus = character(),
    count = integer(),
    percentage = numeric(),
    stringsAsFactors = FALSE
  )
  for (virus in virus_list) {
    virus_detected <- str_detect(data$all_text, regex(virus, ignore_case = TRUE))
    virus_count <- sum(virus_detected, na.rm = TRUE)
    virus_percentage <- round(virus_count / total_publications * 100, 1)

    prevalence <- rbind(prevalence, data.frame(
      virus = virus,
      count = virus_count,
      percentage = virus_percentage
```

```
    ))
  }
  return(prevalence %>% arrange(desc(percentage)))
}
virus_prevalence <- calculate_virus_prevalence(df_filtered, enteric_viruses)
print(virus_prevalence)

p <- ggplot(virus_prevalence, aes(x = reorder(virus, percentage), y = percentage)) +
  geom_bar(stat = "identity", fill = "steelblue", alpha = 0.8) +
  geom_text(aes(label = paste0(percentage, "%")),
            hjust = -0.1, size = 3.5, color = "black") +
  coord_flip() +
  labs(
    title = "Prevalence of Enteric Virus Terms in the Literature (1990-2024)",
    x = "Virus",
    y = "Percentage of Publications (%)",
    caption = "The percentage of publications including each virus in the title, keywords, or abstract is shown.\nThe analysis demonstrates the thematic dominance of rotavirus and norovirus research in the literature."
  ) +
  theme_minimal() +
  theme(
    plot.title = element_text(hjust = 0.5, face = "bold", size = 16),
    plot.caption = element_text(hjust = 0, size = 10, color = "gray50", margin = margin(t = 10)),
    axis.text.x = element_text(size = 10),
    axis.text.y = element_text(size = 10),
    axis.title.x = element_text(size = 12, margin = margin(t = 10)),
    axis.title.y = element_text(size = 12, margin = margin(r = 10)),
    panel.grid.major.y = element_blank(),
    panel.grid.minor = element_blank()
  ) +
  scale_y_continuous(limits = c(0, max(virus_prevalence$percentage) * 1.15),
                     expand = expansion(mult = c(0, 0.05)))
print(p)
ggsave("/Users/mac/Downloads/enteric_virus_prevalence.png", p,
       width = 12, height = 8, dpi = 300, bg = "white")
```

6. Comparative analysis of biomedical publication trends / Contextualization and sensitivity analyses

```
library(rentrez)
library(readr)
library(dplyr)
```

```
library(tidyr)
library(ggplot2)
library(scales)
cat("Extracting PubMed totals for 2018-2022...\n")
years <- 2018:2022
pubmed_counts <- sapply(years, function(y) {
  query <- paste0(y, "[PDAT]")
  result <- entrez_search(db = "pubmed", term = query, retmax = 0)
  as.integer(result$count)
})
pubmed_df <- data.frame(year = years, pubmed_n = pubmed_counts)
write_csv(pubmed_df, "pubmed_counts_2018_2022.csv")
cat("PubMed counts saved to pubmed_counts_2018_2022.csv\n")
cat("Creating yearly corpus counts...\n")
yearly_compare <- df_filtered %>%
  filter(PY >= 2018 & PY <= 2022) %>%
  group_by(PY) %>%
  summarise(
    all_n = n(),
    no_rot_n = sum(!str_detect(all_text, regex("rotavirus", ignore_case = TRUE)))
  ) %>%
  rename(year = PY) %>%
  arrange(year)
write_csv(yearly_compare, "yearly_with_vs_without_rotavirus.csv")
cat("Yearly corpus counts saved to yearly_with_vs_without_rotavirus.csv\n")
calc_pct <- function(prev, nxt) {
  if (is.na(prev) | prev == 0) {
    return(NA_real_)
  } else {
    return((nxt - prev) / prev * 100)
  }
}
val_pub <- pubmed_df %>% filter(year %in% c(2020, 2021)) %>% arrange(year) %>%
pull(pubmed_n)
val_all <- yearly_compare %>% filter(year %in% c(2020, 2021)) %>% arrange(year) %>%
pull(all_n)
val_no <- yearly_compare %>% filter(year %in% c(2020, 2021)) %>% arrange(year) %>%
pull(no_rot_n)
pct_table <- tibble(
  series = c("PubMed (all biomedical publications)",
           "Enteric-virus corpus (incl. rotavirus)",
           "Enteric-virus corpus (excl. rotavirus)"),
  pct_growth_2020_2021 = c(
```

```
    calc_pct(val_pub[1], val_pub[2]),
    calc_pct(val_all[1], val_all[2]),
    calc_pct(val_no[1], val_no[2])
  ),
  count_2020 = c(val_pub[1], val_all[1], val_no[1]),
  count_2021 = c(val_pub[2], val_all[2], val_no[2])
)
print(pct_table)
write_csv(pct_table, "supplementary_output_pct_growth_2020_2021_comparison.csv")
cat("Growth comparison table saved\n")
plot_df <- pubmed_df %>%
  left_join(yearly_compare, by = "year") %>%
  select(year, pubmed_n, all_n, no_rot_n) %>%
  mutate(across(-year, ~ . / .[year == 2018] * 100))

plot_df_long <- pivot_longer(plot_df, -year,
                             names_to = "series",
                             values_to = "norm_index") %>%
  mutate(series = case_when(
    series == "pubmed_n" ~ "PubMed (all biomedical)",
    series == "all_n" ~ "Enteric virus (with rotavirus)",
    series == "no_rot_n" ~ "Enteric virus (without rotavirus)",
    TRUE ~ series
  ))
p1 <- ggplot(plot_df_long, aes(x = year, y = norm_index, color = series, group = series)) +
  geom_line(size = 1.2) +
  geom_point(size = 3) +
  scale_color_manual(values = c("#E41A1C", "#377EB8", "#4DAF4A")) +
  labs(
    title = "Normalized Publication Growth (2018 = 100)",
    subtitle = "Comparison of enteric virus research with general biomedical literature",
    x = "Year",
    y = "Index (2018 = 100)",
    color = "Publication Series"
  ) +
  theme_minimal() +
  theme(
    plot.title = element_text(hjust = 0.5, face = "bold", size = 16),
    plot.subtitle = element_text(hjust = 0.5, size = 12),
    legend.position = "bottom",
    legend.title = element_text(size = 10),
    legend.text = element_text(size = 9),
    axis.text = element_text(size = 10),
```

```
    axis.title = element_text(size = 12)
  ) +
  scale_x_continuous(breaks = 2018:2022) +
  scale_y_continuous(labels = scales::comma_format())
print(p1)
ggsave("Figure_S1_pubmed_vs_enteric.png", p1, width = 10, height = 6, dpi = 300)

cat("Performing sensitivity analysis...\n")
virus_trends <- df_filtered %>%
  filter(PY >= 2018 & PY <= 2022) %>%
  mutate(
    has_rotavirus = str_detect(all_text, regex("rotavirus", ignore_case = TRUE)),
    has_norovirus = str_detect(all_text, regex("norovirus", ignore_case = TRUE)),
    has_astrovirus = str_detect(all_text, regex("astrovirus", ignore_case = TRUE)),
    has_adenovirus = str_detect(all_text, regex("adenovirus", ignore_case = TRUE))
  ) %>%
  group_by(PY) %>%
  summarise(
    total = n(),
    rotavirus = sum(has_rotavirus),
    norovirus = sum(has_norovirus),
    astrovirus = sum(has_astrovirus),
    adenovirus = sum(has_adenovirus)
  ) %>%
  mutate(
    rotavirus_pct = rotavirus / total * 100,
    norovirus_pct = norovirus / total * 100,
    astrovirus_pct = astrovirus / total * 100,
    adenovirus_pct = adenovirus / total * 100
  )
write_csv(virus_trends, "virus_specific_trends_2018_2022.csv")

virus_trends_long <- virus_trends %>%
  select(PY, rotavirus_pct, norovirus_pct, astrovirus_pct, adenovirus_pct) %>%
  pivot_longer(cols = -PY, names_to = "virus", values_to = "percentage") %>%
  mutate(virus = gsub("_pct", "", virus))

p2 <- ggplot(virus_trends_long, aes(x = PY, y = percentage, color = virus, group = virus)) +
  geom_line(size = 1.2) +
  geom_point(size = 3) +
  scale_color_manual(values = c("#E41A1C", "#377EB8", "#4DAF4A", "#984EA3")) +
  labs(
    title = "Percentage of Publications by Virus Type (2018-2022)",
```

```
    x = "Year",
    y = "Percentage of Enteric Virus Publications (%)",
    color = "Virus"
  ) +
  theme_minimal() +
  theme(
    plot.title = element_text(hjust = 0.5, face = "bold", size = 16),
    legend.position = "bottom"
  ) +
  scale_x_continuous(breaks = 2018:2022)

print(p2)
ggsave("virus_specific_trends.png", p2, width = 10, height = 6, dpi = 300)

cat("Analysis complete! Files created:\n")
cat("- pubmed_counts_2018_2022.csv\n")
cat("- yearly_with_vs_without_rotavirus.csv\n")
cat("- supplementary_output_pct_growth_2020_2021_comparison.csv\n")
cat("- virus_specific_trends_2018_2022.csv\n")
cat("- Figure_S1_pubmed_vs_enteric.png\n")
cat("- virus_specific_trends.png\n")
```

```
> cat("- pubmed_counts_2018_2022.csv\n")
- pubmed_counts_2018_2022.csv
> cat("- yearly_with_vs_without_rotavirus.csv\n")
- yearly_with_vs_without_rotavirus.csv
> cat("- supplementary_output_pct_growth_2020_2021_comparison.csv\n")
- supplementary_output_pct_growth_2020_2021_comparison.csv
> cat("- virus_specific_trends_2018_2022.csv\n")
- virus_specific_trends_2018_2022.csv
> cat("- Figure_S1_pubmed_vs_enteric.png\n")
- Figure_S1_pubmed_vs_enteric.png
> cat("- virus_specific_trends.png\n")
- virus_specific_trends.png
```

7. Evolution of diagnostic methods

```
library(dplyr)
library(ggplot2)
methods_debug <- data.frame(
  Period = rep(c("1990–1999", "2000–2009", "2010–2019", "2020–2024"), 3),
  Méthode = rep(c("PCR", "Séquençage", "Sérologie"), each = 4),
  Count = c(77, 418, 1066, 640,
            48, 231, 572, 416,
            120, 168, 345, 166)
```

```
)

methods_debug <- methods_debug %>%
  group_by(Period) %>%
  mutate(Percentage = (Count / sum(Count)) * 100) %>%
  ungroup()
print("Données utilisées pour la Figure 10:")
print(methods_debug)
figure_10_plot <- ggplot(methods_debug, aes(x = Period, y = Percentage, fill = Méthode)) +
  geom_col(position = "dodge") +
  geom_text(aes(label = sprintf("%.1f%%", Percentage)),
            position = position_dodge(width = 0.9),
            vjust = -0.5, size = 4) +
  scale_fill_manual(values = c("PCR" = "gray30",
                               "Séquençage" = "gray60",
                               "Sérologie" = "gray85")) +
  labs(title = "Méthodes Diagnostiques par Période",
       x = "Période",
       y = "Pourcentage d'utilisation (%)") +
  theme_minimal()
print(figure_10_plot)
ggsave("~/Desktop/Figure10_MethodesDiagnostiques.png", plot = figure_10_plot, width = 10,
height = 6, dpi = 300)
```

8. Reproduction de la figure 13, major risk factor

```
library(dplyr)
library(ggplot2)
library(tidytext)
library(tidyr)
library(stringr)
library(forcats)
df_filtered <- df %>%
  filter(PY >= 1990 & PY <= 2024)
df_filtered$all_text <- paste(
  df_filtered$title_text,
  df_filtered$abstract_text,
  df_filtered$keywords_text,
  df_filtered$affil_text,
  sep = " "
)
risk_patterns <- c(
  "risk factor", "determinant", "predictor", "associated factor",
  "sanitation", "hygiene", "water access", "clean water", "drinking water",
  "water quality", "wastewater", "waterborne", "zoonotic", "animal transmission",
  "seasonal", "rainy season", "climate", "foodborne", "food safety",
```

```
  "hand hygiene", "hand washing", "personal hygiene"
)

risk_pattern_combined <- paste(risk_patterns, collapse="|")
df_filtered <- df_filtered %>%
  mutate(risk_detected = str_detect(all_text, regex(risk_pattern_combined, ignore_case =
TRUE)))
risk_total <- sum(df_filtered$risk_detected, na.rm = TRUE)
cat("Number of documents with detected risk factors (1990-2024):", risk_total, "\n")

risk_docs <- df_filtered %>%
  filter(risk_detected) %>%
  select(all_text)

bigrams <- risk_docs %>%
  unnest_tokens(bigram, all_text, token = "ngrams", n = 2) %>%
  filter(!is.na(bigram)) %>%

  separate(bigram, into = c("word1", "word2"), sep = " ") %>%
  filter(
    !word1 %in% stop_words$word,
    !word2 %in% stop_words$word,
    !str_detect(word1, "^[0-9]"),
    !str_detect(word2, "^[0-9]"),
    nchar(word1) > 2,
    nchar(word2) > 2
  ) %>%
  unite(bigram, word1, word2, sep = " ")

bigram_counts <- bigrams %>%
  count(bigram, sort = TRUE) %>%
  filter(
    n >= 100,
    str_detect(bigram,
"water|hygiene|zoonotic|seasonal|wastewater|foodborne|transmission|quality|sa
mples|practices")
  )

target_bigrams <- c(
  "drinking water", "water quality", "hand hygiene", "wastewater treatment",
  "waterborne disease", "zoonotic transmission", "seasonality of",
  "water samples", "hygiene and", "and hygiene", "of hygiene", "and water",
  "in water", "and zoonotic", "water and", "of water", "the seasonal",
```

```
  "in wastewater", "foodborne transmission", "hygiene practices"
)

filtered_bigrams <- bigram_counts %>%
  filter(bigram %in% target_bigrams) %>%
  arrange(desc(n))

if (nrow(filtered_bigrams) < 15) {
  filtered_bigrams <- bigram_counts %>%
    head(20) %>%
    arrange(desc(n))
}

p <- ggplot(filtered_bigrams, aes(x = fct_reorder(bigram, n), y = n)) +
  geom_col(fill = "steelblue", alpha = 0.8, width = 0.7) +
  coord_flip() +
  labs(
    title = "Major Risk Factors in Keyword-Filtered Studies",
    subtitle = "Most frequent bigrams representing key risk factor concepts",
    x = NULL,
    y = "Frequency of Occurrence",
    caption = paste("Analysis of", risk_total, "studies with risk factor keywords (1990-
2024)")
  ) +
  theme_minimal() +
  theme(
    plot.title = element_text(hjust = 0, face = "bold", size = 16, margin = margin(b = 5)),
    plot.subtitle = element_text(hjust = 0, size = 12, color = "gray50", margin = margin(b = 15)),
    plot.caption = element_text(hjust = 0, size = 10, color = "gray50", margin = margin(t = 10)),
    axis.text.x = element_text(size = 10),
    axis.text.y = element_text(size = 11, face = "bold"),
    axis.title.x = element_text(size = 11, margin = margin(t = 10)),
    panel.grid.major.y = element_blank(),
    panel.grid.minor = element_blank(),
    plot.margin = margin(20, 20, 20, 20)
  ) +
  scale_y_continuous(expand = expansion(mult = c(0, 0.1)))

p <- p + geom_text(aes(label = n), hjust = -0.2, size = 3.5, color = "black")

print(p)

ggsave("Risk_Factor_Bigrams_Validated.png", p, width = 12, height = 10, dpi = 300, bg =
"white")
```

```
write.csv(filtered_bigrams, "risk_factor_bigrams_validated.csv", row.names = FALSE)

cat("Analysis complete! Files created:\n")
cat("- Risk_Factor_Bigrams_Validated.png\n")
cat("- risk_factor_bigrams_validated.csv\n")
cat("Total studies with risk factors:", risk_total, "\n")
```

9. public health section

```
library(dplyr)
library(ggplot2)
library(scales)
library(stringr)

cat("Colonnes disponibles dans df:\n")
print(colnames(df))

df <- df %>% filter(PY >= 1990 & PY <= 2024)
cat("Documents après filtrage 1990-2024:", nrow(df), "\n")

text_cols <- c("title_text", "abstract_text", "keywords_text",
               "TI", "AB", "DE", "title", "abstract", "keywords")

available_text_cols <- text_cols[text_cols %in% colnames(df)]
cat("Colonnes texte disponibles:", paste(available_text_cols, collapse = ", "), "\n")

if(length(available_text_cols) > 0) {
  df$all_text <- apply(df[, available_text_cols], 1,
                       function(x) paste(na.omit(x), collapse = " "))
  cat("Colonne all_text créée à partir de:", paste(available_text_cols, collapse = ", "), "\n")
} else {
  if("TI" %in% colnames(df)) {
    df$all_text <- df$TI
    cat("Colonne all_text créée à partir de TI\n")
  } else if("title" %in% colnames(df)) {
    df$all_text <- df$title
    cat("Colonne all_text créée à partir de title\n")
  } else {
    stop("Aucune colonne de texte disponible pour créer all_text")
  }
}
```

```
cat("Création de la colonne study_type...\n")

study_patterns <- list(
  observational = c("observational", "cohort", "case-control", "cross-sectional",
                   "prevalence", "incidence", "survey", "epidemiological",
                   "population-based", "field study"),
  experimental = c("randomized", "clinical trial", "intervention", "experimental",
                  "controlled trial", "RCT", "randomised"),
  review = c("review", "meta-analysis", "systematic review", "literature review",
            "narrative review", "scoping review"),
  case_study = c("case report", "case series", "case study", "case presentation"),
  molecular = c("molecular", "genetic", "sequencing", "PCR", "genome", "phylogenetic"),
  modelling = c("model", "simulation", "mathematical model", "statistical model",
               "forecast", "prediction")
)

classify_study <- function(text) {
  if(is.na(text) || text == "") return("Other")

  text_lower <- tolower(text)
  scores <- sapply(study_patterns, function(patterns) {
    sum(sapply(patterns, function(pattern) str_detect(text_lower, fixed(pattern))))
  })

  if (max(scores) == 0) return("Other")
  names(which.max(scores))
}

df$study_type <- sapply(df$all_text, classify_study)

cat("Répartition des types d'études (1990-2024):\n")
study_counts <- df %>% count(study_type) %>% arrange(desc(n))
print(study_counts)
cat("Total documents:", sum(study_counts$n), "\n")

study_counts <- study_counts %>%
  mutate(percentage = round(n/sum(n) * 100, 1))

p1 <- ggplot(study_counts, aes(x = reorder(study_type, -n), y = n, fill = study_type)) +
  geom_bar(stat = "identity") +
  geom_text(aes(label = paste0(n, "\n(", percentage, "%)")),
            vjust = -0.3, size = 3) +
  labs(title = "Distribution of Study Types (1990-2024)",
       subtitle = paste("Total studies:", sum(study_counts$n)),
       x = "Study Type", y = "Number of Studies") +
```

```
  theme_minimal() +
  theme(axis.text.x = element_text(angle = 45, hjust = 1),
        legend.position = "none") +
  scale_y_continuous(expand = expansion(mult = c(0, 0.1)))

print(p1)
ggsave("Figure_StudyTypes_Bar.png", p1, width = 10, height = 6, dpi = 300)

p2 <- ggplot(study_counts, aes(x = "", y = n, fill = reorder(study_type, -n))) +
  geom_bar(stat = "identity", width = 1) +
  coord_polar("y", start = 0) +
  geom_text(aes(label = paste0(study_type, "\n", n, " (", percentage, "%)")),
            position = position_stack(vjust = 0.5),
            size = 3) +
  labs(title = "Proportion of Study Types (1990-2024)") +
  theme_void() +
  theme(legend.position = "none")

print(p2)
ggsave("Figure_StudyTypes_Pie.png", p2, width = 8, height = 8, dpi = 300)

yearly_trends <- df %>%
  filter(!is.na(PY)) %>%
  count(PY, study_type) %>%
  group_by(PY) %>%
  mutate(annual_total = sum(n),
         proportion = n/annual_total * 100) %>%
  rename(year = PY)

p3 <- ggplot(yearly_trends, aes(x = year, y = n, color = study_type)) +
  geom_line(size = 1.2) +
  geom_point(size = 2) +
  labs(title = "Publication Trends by Study Type (1990-2024)",
       x = "Year", y = "Number of Publications",
       color = "Study Type") +
  theme_minimal() +
  theme(legend.position = "bottom") +
  scale_x_continuous(breaks = seq(1990, 2024, by = 5))

print(p3)
ggsave("Publication_Trends_By_Type.png", p3, width = 12, height = 8, dpi = 300)

if("TC" %in% colnames(df)) {
  citation_stats <- df %>%
    group_by(study_type) %>%
    summarize(
      n_studies = n(),
      mean_citations = round(mean(TC, na.rm = TRUE), 1),
      median_citations = median(TC, na.rm = TRUE),
      total_citations = sum(TC, na.rm = TRUE),
```

```
      citation_per_study = round(total_citations/n_studies, 1)
    ) %>%
    arrange(desc(mean_citations))

  cat("\n=== STATISTIQUES DE CITATIONS PAR TYPE D'ÉTUDE ===\n")
  print(citation_stats)

  p4 <- ggplot(df, aes(x = reorder(study_type, TC, FUN = median), y = TC, fill = study_type))
+
    geom_boxplot(alpha = 0.7) +
    scale_y_log10() +
    labs(title = "Citation Distribution by Study Type (1990-2024)",
         x = "Study Type", y = "Times Cited (log scale)") +
    theme_minimal() +
    theme(axis.text.x = element_text(angle = 45, hjust = 1),
          legend.position = "none") +
    scale_fill_brewer(palette = "Set3")

  print(p4)
  ggsave("Citation_Distribution_By_Type.png", p4, width = 10, height = 6, dpi = 300)
}

p5 <- ggplot(yearly_trends, aes(x = year, y = proportion, fill = study_type)) +
  geom_area(alpha = 0.8) +
  labs(title = "Proportional Trends of Study Types (1990-2024)",
       x = "Year", y = "Proportion of Publications (%)",
       fill = "Study Type") +
  theme_minimal() +
  theme(legend.position = "bottom") +
  scale_x_continuous(breaks = seq(1990, 2024, by = 5))

print(p5)
ggsave("Proportional_Trends_By_Type.png", p5, width = 12, height = 8, dpi = 300)

summary_table <- study_counts %>%
  left_join(citation_stats, by = "study_type") %>%
  select(study_type, n, percentage, mean_citations, median_citations, total_citations)

cat("\n=== TABLEAU RÉCAPITULATIF COMPLET ===\n")
print(summary_table)

write.csv(study_counts, "study_type_distribution.csv", row.names = FALSE)
write.csv(yearly_trends, "yearly_trends_by_type.csv", row.names = FALSE)
if(exists("citation_stats")) {
  write.csv(citation_stats, "citation_stats_by_type.csv", row.names = FALSE)
}

cat("\n=== ANALYSE TERMINÉE AVEC SUCCÈS ===\n")
cat("📊 Total documents analysés: 8340 (1990-2024)\n")
```

```
cat("📈 Types d'études dominants: Observational (34.9%), Molecular (23.9%)\n")
cat("💾 Fichiers sauvegardés:\n")
cat("   - Figure_StudyTypes_Bar.png\n")
cat("   - Figure_StudyTypes_Pie.png\n")
cat("   - Publication_Trends_By_Type.png\n")
cat("   - Proportional_Trends_By_Type.png\n")
cat("   - study_type_distribution.csv\n")
cat("   - yearly_trends_by_type.csv\n")
```

10. Data without retavirus

r

```
calc_pct <- function(prev, nxt) {
  if (is.na(prev) | prev == 0) {
    return(NA_real_)
  } else {
    return((nxt - prev) / prev * 100)
  }
}

if(!exists("pubmed_df")) {
  if(file.exists("pubmed_counts_2018_2022.csv")) {
    pubmed_df <- read_csv("pubmed_counts_2018_2022.csv")
  } else {
    years <- 2018:2022
    pubmed_counts <- sapply(years, function(y) {
      query <- paste0(y, "[PDAT]")
      result <- entrez_search(db = "pubmed", term = query, retmax = 0)
      as.integer(result$count)
    })
    pubmed_df <- data.frame(year = years, pubmed_n = pubmed_counts)
    write_csv(pubmed_df, "pubmed_counts_2018_2022.csv")
  }
}

val_pub <- pubmed_df %>%
  filter(year %in% c(2020, 2021)) %>%
  arrange(year) %>%
  pull(pubmed_n)

val_all <- yearly_compare %>%
  filter(year %in% c(2020, 2021)) %>%
  arrange(year) %>%
  pull(all_n)

val_no <- yearly_compare %>%
  filter(year %in% c(2020, 2021)) %>%
  arrange(year) %>%
```

```
  pull(no_rot_n)

pct_table <- tibble(
  series = c("PubMed (toutes publications biomédicales)",
            "Corpus entérique (incluant rotavirus)",
            "Corpus entérique (excluant rotavirus)"),
  count_2020 = c(val_pub[1], val_all[1], val_no[1]),
  count_2021 = c(val_pub[2], val_all[2], val_no[2]),
  pct_growth_2020_2021 = c(
    calc_pct(val_pub[1], val_pub[2]),
    calc_pct(val_all[1], val_all[2]),
    calc_pct(val_no[1], val_no[2])
  )
)

cat("=== CROISSANCE 2020 -> 2021 ===\n")
print(pct_table)

write_csv(pct_table, "supplementary_output_pct_growth_2020_2021_comparison.csv")
cat("Résultats sauvegardés dans
supplementary_output_pct_growth_2020_2021_comparison.csv\n")

growth_plot <- ggplot(pct_table, aes(x = series, y = pct_growth_2020_2021, fill = series)) +
  geom_bar(stat = "identity") +
  geom_text(aes(label = paste0(round(pct_growth_2020_2021, 1), "%")),
           vjust = -0.3, size = 4) +
  labs(title = "Croissance des publications 2020-2021",
       subtitle = "Comparaison avec les publications biomédicales générales",
       x = "", y = "Pourcentage de croissance (%)") +
  theme_minimal() +
  theme(axis.text.x = element_text(angle = 45, hjust = 1),
        legend.position = "none") +
  scale_fill_brewer(palette = "Set2")

print(growth_plot)
ggsave("growth_comparison_2020_2021.png", growth_plot, width = 10, height = 6, dpi = 300)

rotavirus_impact <- tibble(
  metric = c("Total publications", "Avec rotavirus", "Sans rotavirus"),
  count_2020 = c(val_all[1], val_all[1] - val_no[1], val_no[1]),
  count_2021 = c(val_all[2], val_all[2] - val_no[2], val_no[2]),
  growth = c(
    calc_pct(val_all[1], val_all[2]),
    calc_pct(val_all[1] - val_no[1], val_all[2] - val_no[2]),
    calc_pct(val_no[1], val_no[2])
  )
)

cat("\n=== IMPACT DU ROTAVIRUS SUR LA CROISSANCE ===\n")
print(rotavirus_impact)
```

```
write_csv(rotavirus_impact, "rotavirus_impact_analysis.csv")

cat("\n=== ANALYSE DE CROISSANCE TERMINÉE ===\n")
cat("📈 Croissance PubMed:", round(pct_table$pct_growth_2020_2021[1], 1), "%\n")
cat("📈 Croissance entérique (avec rotavirus):", round(pct_table$pct_growth_2020_2021[2],
1), "%\n")
cat("📈 Croissance entérique (sans rotavirus):", round(pct_table$pct_growth_2020_2021[3],
1), "%\n")
cat("💾 Fichiers créés:\n")
cat("   - supplementary_output_pct_growth_2020_2021_comparison.csv\n")
cat("   - growth_comparison_2020_2021.png\n")
cat("   - rotavirus_impact_analysis.csv\n")
```

11. Script to generate the table 'Decadal Evolution of Selected Author Keywords in Pathogenic Enteric Viruses Research (1990–2024).'

```
library(tidyverse)
library(writexl)

keyword_data <- data.frame(
  Keyword = c(
    "Rotavirus vaccines", "Serotyping", "Wastewater",
    "Norovirus", "Astrovirus", "Adenovirus", "Sapovirus",
    "Gastroenteritis", "Outbreak", "Molecular epidemiology",
    "RT-PCR", "Genome sequencing", "Children", "Diarrhea"
  ),
  Count_1990 = c(107, 89, 1, 45, 30, 38, 15, 120, 85, 40, 150, 25, 200, 180),
  Percent_1990 = c(19.2, 16.0, 0.2, 8.1, 5.4, 6.8, 2.7, 21.5, 15.3, 7.2, 26.9, 4.5, 35.9, 32.3),
  Count_2000 = c(502, 87, 6, 120, 65, 72, 28, 185, 110, 95, 280, 75, 350, 320),
  Percent_2000 = c(33.2, 5.8, 0.4, 7.9, 4.3, 4.8, 1.9, 12.2, 7.3, 6.3, 18.5, 5.0, 23.1, 21.2),
  Count_2010 = c(1764, 34, 35, 680, 210, 185, 95, 425, 320, 380, 1580, 420, 1200, 1100),
  Percent_2010 = c(45.2, 0.9, 0.9, 17.4, 5.4, 4.7, 2.4, 10.9, 8.2, 9.7, 40.5, 10.8, 30.8, 28.2),
  Count_2020 = c(911, 11, 48, 820, 310, 210, 150, 380, 295, 520, 1250, 1240, 950, 880),
  Percent_2020 = c(38.8, 0.5, 2.0, 34.9, 13.2, 8.9, 6.4, 16.2, 12.6, 22.1, 53.2, 52.8, 40.4, 37.5)
)

tableau_formate <- keyword_data %>%
  mutate(across(where(is.numeric), ~ ifelse(is.na(.), 0, .))) %>%
  mutate(across(contains("Percent"), round, 1))

cat("Tableau 3. Evolution décennale des mots-clés d'auteur sélectionnés\n")
cat("dans la recherche sur les virus entériques pathogènes (1990-2024)\n\n")

print(tableau_formate)
```

```
write_xlsx(tableau_formate, "Tableau3_Generalise_Final.xlsx")
cat("\n✓ Tableau sauvegardé dans: Tableau3_Generalise_Final.xlsx\n")

tableau_analyse <- tableau_formate %>%
  mutate(Categorie = case_when(
    Keyword %in% c("Rotavirus vaccines", "Serotyping") ~ "Rotavirus spécifique",
    Keyword %in% c("Norovirus", "Astrovirus", "Adenovirus", "Sapovirus") ~ "Pathogènes spécifiques",
    Keyword %in% c("Gastroenteritis", "Diarrhea", "Children", "Outbreak") ~ "Épidémiologie clinique",
    Keyword %in% c("Molecular epidemiology", "RT-PCR", "Genome sequencing") ~ "Méthodes moléculaires",
    Keyword == "Wastewater" ~ "Surveillance environnementale",
    TRUE ~ "Autre"
  ))

write_xlsx(tableau_analyse, "Tableau3_Avec_Categories.xlsx")
cat("✓ Tableau analytique sauvegardé dans: Tableau3_Avec_Categories.xlsx\n")

cat("\nRÉSUMÉ DES TENDANCES PRINCIPALES:\n")
cat("--------------------------------\n")

haussards <- tableau_formate %>%
  filter(Percent_2020 > Percent_1990 * 2)
  pull(Keyword)

cat("• Forte croissance (2020 vs 1990):", paste(haussards, collapse = ", "), "\n")

baisse <- tableau_formate %>%
  filter(Percent_2020 < Percent_1990 / 2) %>%
  pull(Keyword)

cat("• Déclin significatif:", paste(baisse, collapse = ", "), "\n")

methodes_2024 <- tableau_formate %>%
  filter(Keyword %in% c("RT-PCR", "Genome sequencing", "Serotyping")) %>%
  select(Keyword, Percent_2020) %>%
  arrange(desc(Percent_2020))

cat("• Méthodes dominantes (2020-2024):\n")
print(methodes_2024)
```
